# The importance of a charge transfer descriptor for screening potential CO_2_ reduction electrocatalysts

**DOI:** 10.1038/s41467-023-37929-4

**Published:** 2023-05-05

**Authors:** Stefan Ringe

**Affiliations:** Department of Chemistry, Korea University, Seoul, 02841 Republic of Korea

**Keywords:** Electrocatalysis, Catalytic mechanisms, Density functional theory, Materials for energy and catalysis

## Abstract

It has been over twenty years since the linear scaling of reaction intermediate adsorption energies started to coin the fields of heterogeneous and electrocatalysis as a blessing and a curse at the same time. It has established the possibility to construct activity volcano plots as a function of a single or two readily accessible adsorption energies as descriptors, but also limited the maximal catalytic conversion rate. In this work, it is found that these established adsorption energy-based descriptor spaces are not applicable to electrochemistry, because they are lacking an important additional dimension, the potential of zero charge. This extra dimension arises from the interaction of the electric double layer with reaction intermediates which does not scale with adsorption energies. At the example of the electrochemical reduction of CO_2_ it is shown that the addition of this descriptor breaks the scaling relations, opening up a huge chemical space that is readily accessible via potential of zero charge-based material design. The potential of zero charge also explains product selectivity trends of electrochemical CO_2_ reduction in close agreement with reported experimental data highlighting its importance for electrocatalyst design.

## Abstract (web summary)

The electric double layer controls the product selectivity and conversion rate of electrochemical processes. Here the author presents a double layer-corrected computational electrocatalyst screening approach using CO2 reduction as an example that highlights the importance of the potential of zero charge descriptor.

## Introduction

Over the last years, computational, *first-principles*-based simulations have become a key tool in the global thrive towards a sustainable energy landscape. Density Functional Theory (DFT) has become in particular in heterogeneous surface catalysis a valuable means to explore reaction pathways and evaluate the catalytic ability of specific materials and active sites^1^. Unfortunately, complex reaction pathways often occur which require the computation of a large number of intermediate’s formation energies and reaction barriers, making it hard to utilize this approach for high-throughput screening of catalysts. The discovery of an often linear correlation of intermediate’s adsorption energies^2,3^, known as *scaling relations*^4^ has been thus a breakthrough in reducing the dimensionality of the catalytic activity space. In combination with Brønsted-Evans-Polanyi (BEP) relations^5–8^ which resemble the often linear correlations of activation energies with intermediate adsorption energies, this results in activity curves as a function of a single or two adsorption energies. According to the Sabatier principle^9,10^ these take the form of activity volcanos with the best catalyst binding the descriptor intermediate neither too weak nor too strong^11^. This has allowed the high-throughput screening of catalysts^12–15^ and a simplified discussion of experimental findings by a single or two adsorption energies^16^. On the downside, the scaling relations have made it impossible to independently vary the kinetic barriers of all elementary steps which would be needed to achieve the optimal catalyst^2,3^. The breaking of scaling relations to go beyond the dimensionality and thus limiting activity shown in the activity volcano has therefore been a key obstacle for the development of more active solid catalysts^17,18^.

In contrast to thermocatalysis, electrocatalysis involves the transfer of electrons and protons which electrochemical potentials needs to be calculated. The Computational Hydrogen Electrode (CHE) approach considers entirely proton-coupled electron transfer (PCET) steps and derives these electrochemical potentials simply from reference to the Standard Hydrogen Electrode (SHE) at which these are in equilibrium with Hydrogen gas^19,20^. By again considering scaling and BEP relations for PCET steps^21^, the full potential and pH dependence of all intermediate’s formation energies is accessible from often only a single or two formation energies^22^. Again, this descriptor-based approach has been used from high-throughput screening using machine learning^23–25^ or interpretation of experimental findings^22^.

Due to the origination of this approach from surface science, the formation energies of reaction intermediates even in electrochemistry were calculated on simple solid surface-vacuum model systems. Recently, this simplistic picture has been increasingly doubted, mostly due to the neglect of the electric double layer^26,27^. At applied potentials away from the Potential of Zero Charge (PZC), all metal electrodes build up surface charge density which is compensated by counter charge in the solution creating a significant interfacial electric double layer field. This surface charge density and the corresponding electric field have been shown to strongly affect the stability of key reaction intermediates and with that the catalyst’s performance and product selectivity^28–35^. The electric double layer effect is particularly critical in the electrochemical reduction of CO_2_ (CO_2_RR). Here, the formation of intermediates like ^*^CO_2_ and ^*^OCCO is rate-limiting which gives rise to a significant electric dipole that is stabilized by the surrounding double layer field^28–30,32^. Despite this recently discovered significance of the electric double layer, a correspondingly corrected descriptor-picture or activity volcano has not been derived so far. Instead, the community has mostly relied on the surface chemical approach resulting in one-dimensional (e.g. CO adsorption energy in CO_2_RR to CO^36^) or two-dimensional (e.g. C and CO adsorption energies for CO_2_RR to higher reduced products^37,38^) activity volcano plots.

In this work, I develop a descriptor-based activity model for CO_2_RR to CO with an explicit account for the electrochemical interface. The focus on CO_2_RR is due to two reasons. First, previous studies have revealed significant electric double-layer effects, stressing the urgency of this extension for the reliable screening of electrocatalysts. Secondly, electrochemical CO_2_RR has recently emerged as an attractive prospect for a sustainable carbon cycle and energy society. The focus on CO is due to the economical relevance of this process^39^ and the simple and well-established reaction mechanism allowing for a more reliable kinetic model. From this, it is revealed that the PZC and CO formation energies as the relevant descriptors and also discover that the same descriptors can be also quite generally used to discuss product selectivity of electrochemical CO_2_RR. Thus the descriptor space might be used to new CO_2_RR electrocatalysts for a defined product. This work makes a significant step forward in the screening of electrocatalysts and opens design avenues based on the PZC of materials that have been neglected so far.

## Results

### Development of descriptor space

**Fig. 1:**
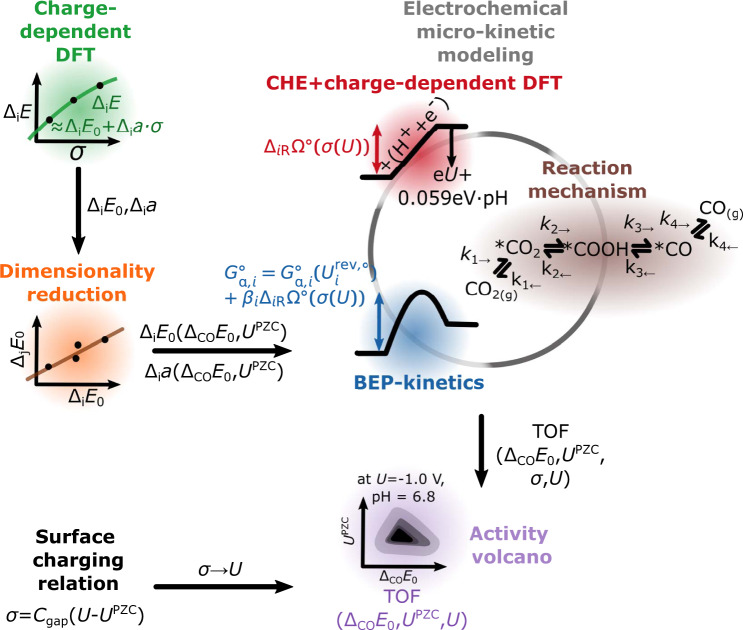
Schematic illustration of the derivation of electrochemical activity volcanos. DFT calculations provide adsorption energies on a chosen facet as a function of surface charge density which can be interpolated. As a result, adsorption energies are obtained at zero surface charge density and the charge sensitivity parameter, and the process is repeated for various facets to generate a database. Then, the data is used to search for correlations among the properties to reduce the dimensionality of descriptors needed to define all adsorption energies and charge sensitivity parameters and thus the full free energy diagram. After this, the determined descriptors CO adsorption energy and PZC are used to build up a two-dimensional TOF figure. For this, the micro-kinetic rate equations are solved on a grid of points on this two-dimensional figure where each point defines a descriptor pair that uniquely links to a full free energy diagram via the determined correlations. To get the potential- and pH dependence of the free energy diagram at a given point, the CHE model is used together with the surface charging relation which eliminates the surface charge density from the expression. Kinetics are described by Brønsted-Evans-Polanyi (BEP) scaling relations with empirically chosen parameters.

Figure 1 schematically illustrates the workflow for deriving an electric double layer-corrected CO_2_RR to CO activity volcano, in this work exemplarily for metallic electrocatalysts. I briefly summarize the procedure here and direct the interested reader to the Methods section A for more details. The electrochemical reduction of CO_2_ to CO is considered to follow the well-established mechanism ^30,40,41^:

1 $$\begin{array}{rcl}{{{{{{{{\rm{CO}}}}}}}}}_{2}({{{{{{{\rm{g}}}}}}}}){+}^{*}&\rightleftharpoons &{}^{*}{{{{{{{{\rm{CO}}}}}}}}}_{2}\\ {}^{*}{{{{{{{{\rm{CO}}}}}}}}}_{2}+{{{{{{{{\rm{H}}}}}}}}}^{+}+{{{{{{{{\rm{e}}}}}}}}}^{-}&\rightleftharpoons &{}^{*}{{{{{{{\rm{COOH}}}}}}}}\\ {}^{*}{{{{{{{\rm{COOH}}}}}}}}+{{{{{{{{\rm{H}}}}}}}}}^{+}+{{{{{{{{\rm{e}}}}}}}}}^{-}&\rightleftharpoons &{}^{*}{{{{{{{\rm{CO}}}}}}}}+{{{{{{{{\rm{H}}}}}}}}}_{2}{{{{{{{\rm{O}}}}}}}}({{{{{{{\rm{l}}}}}}}})\\ {}^{*}{{{{{{{\rm{CO}}}}}}}}&\rightleftharpoons &{{{{{{{\rm{CO}}}}}}}}({{{{{{{\rm{g}}}}}}}})\end{array}$$

The formation/adsorption energy Δ_*i*_Ω of each reaction intermediate *i* is calculated as (see Methods section A for a derivation):

2a $${\Delta }_{i}\Omega (U,pH)=	 \underbrace{{\Delta }_{i}E(\sigma (U))}_{\approx {\Delta }_{i}{E}_{0}+{\Delta }_{i}a\cdot \sigma (U)\,(+{\Delta }_{i}b\cdot \sigma {(U)}^{2})}\\ 	+{\Delta }_{i}{E}^{{{{{{{{\rm{T}}}}}}}},{{{{{{{\rm{S}}}}}}}},{{{{{{{\rm{ZPE}}}}}}}},\circ }+eU+0.0592\cdot pH$$

2b $$\sigma (U)={C}_{{{{{{{{\rm{gap}}}}}}}}}\cdot (U-{U}^{{{{{{{{\rm{PZC}}}}}}}}}),$$

with the surface charge density *σ*, the electrode potential vs. SHE *U*, the zero-point energy (ZPE) and finite temperature correction term Δ_*i*_*E*^T,S,ZPE,∘^, the gap or Helmholtz capacitance *C*_gap_ and the potential of zero charge (PZC) *U*^PZC^. Δ_*i*_*E*(*σ*(*U*)) is the DFT calculated expression, the surface-charge dependent adsorption energy of a specific reaction intermediate relative to the bare slab and CO_2_(g) (in the case of kinetic modeling or reference to the ^*^COOH and ^*^CO_2 _adsorption energies) or CO(g) (in the case of reference to the ^*^CO adsorption energy).

Within DFT, the effect of surface charge is included by means of an implicit solvation model and planar counter charge approach, and a quadratic fit was considered first^29,30,42^. The results for adsorption of the three reaction intermediates on various metallic facets are summarized in Supplementary Materials Table S1. The quadratic contribution to Δ_*i*_*E* related to the quadratic coefficient Δ_*i*_*b* turns out to be at least an order of magnitude smaller than the linear contribution related to the coefficient Δ_*i*_*a* (cf. Supplementary Materials Fig. S5), making Δ_*i*_*E*(*σ*) linear to a good approximation (corresponding to a constant capacitance during adsorption^43^). The quadratic term is thus dropped in the following. From a physical point of view, the charge sensitivity parameter Δ_*i*_*a* is proportional to the electric dipole moment change during adsorption:

3 $${\Delta }_{i}\mu={\varepsilon }^{0}{\varepsilon }^{{{{{{{{\rm{gap}}}}}}}}}{\Delta }_{i}a,$$

with the vacuum permittivity *ε*^0^, the dielectric permittivity of the Helmholtz layer *ε*^gap^ and the change of the electric dipole moment during adsorption Δ_*i*_*μ*. Changes in the electric dipole moment are triggered by charge transfer between adsorbate and surface^26,43–46^. The charge sensitivity parameter for CO_2_ adsorption, and thus the generated dipole moment during adsorption, is a factor of ≈3–10 larger than the coefficients for *COOH and *CO formation (Supplementary Materials Table S1). This is due to the strong interfacial dipole moment created during CO_2_ adsorption which is stabilized by the surrounding electric double-layer field^29,30^. The remaining potential- and pH terms in Eq. (2a) stem from the CHE expression for the thermodynamic energy of a PCET ^19,30^.

Finally, Eq. (2b) denotes the surface charging relation or equation of state, for which again a plate capacitor was assumed. In this expression, the gap capacitance is taken to be constant across metals (≈20 μF/cm^2^)^47–49^ and the PZC which is well known to be proportional to the work function or Fermi level^26,50^, is derived from the calculated Fermi level of each surface plus a constant offset of 4.92 V obtained from fitting the Fermi level to the experimental PZC data (cf. Supplementary Materials Fig. S1). From the energy expression above, all formation energies are determined by three material-specific parameters for each intermediate, the formation energy at the PZC, Δ_*i*_*E*_0_, the parameters measuring the sensitivity of the intermediate with respect to surface charging Δ_*i*_*a*, and the PZC *U*^PZC^. The ZPE and finite temperature corrections are considered roughly independent of the surface^51^.

To further reduce the nine-dimensional descriptor space of the three intermediates which determine the free energy landscape, linear correlations are searched for within this space. First, all properties are correlated with only the CO adsorption energy Δ_CO_*E*_0_. In agreement with the literature^33,52^ a good linear scaling with the *COOH formation energy Δ_COOH_*E*_0_ (*R*^2^ = 0.90) is obtained (Supplementary Materials Fig. S2). The prediction of the *CO_2_ adsorption energy in contrast is significantly worse with a reduced *R*^2^ = 0.72. Most critically, however, the remaining charge sensitivity parameter Δ_*i*_*a* turns out to be not predictable using the CO adsorption energies since no linear correlation can be identified. In addition, the charge sensitivity parameters of *CO and *COOH are found to be uncorrelated (Supplementary Materials Fig. S3) which according to Eq. (2a) means that Δ_CO_Ω and Δ_COOH_Ω are also not correlated. This suggests a breaking of the well-established *COOH-*CO scaling relation^33,52^, meaning that even without the presence of the *CO_2_ intermediate deviations from a one-dimensional activity volcano for CO_2_ reduction to CO are to be expected due to surface charging.

As explained above, Δ_*i*_*a* relates to the amount of charge transferred from the metal surface to the adsorbate during the adsorption process. The charge is transferred from the Fermi level of the metallic slab to the adsorbate. Due to this, the Fermi level seems like an intuitive choice for predicting the qualitative trends of charge transfer and thus Δ_*i*_*a* across metals. Indeed, the work function which correlates with the PZC trends across metals^50,53^, has been already been discussed as an important parameter for CO_2_RR to CO^54^ and the hydrogen evolution reaction (HER)^55,56^. Intriguingly, the inclusion of this descriptor in a two-dimensional multi-regression model happens to increase all *R*^2^ values of adsorption energies and charge sensitivity parameters significantly to 0.9–1.0, with only the charge sensitivity parameter of *CO_2_ being not perfectly scalable at *R*^2^ = 0.76 (Supplementary Materials Fig. S2, S4 and Table S2). The larger uncertainty is likely related to the instability of the ^*^CO_2_ state on many surfaces which requires extrapolating the energy to smaller charges using a fixed high charge geometry^29^. This extrapolation procedure bears some numerical error which might actually be canceled out to some degree by the linear scaling model. I also note that a correlation between adsorption dipole (and thus Δ_*i*_*a*) and PZC has been noticed by a different work recently^46^. Δ_*i*_*a* has, however, also a strong dependence on the adsorption site as well^29^, which might be in parts included in the other CO adsorption energy descriptor, as suggested by the strong linear partial correlation of the charging parameter of CO with the CO adsorption energy (cf. Supplementary Materials Fig. S4 and Table S2).

In sum, the two-dimensional descriptor space spanned by CO adsorption energy and Fermi level gives a reliable representation of the full-dimensional free energy landscape for all considered metal surfaces. Note that this includes both planar surface facets, but also stepped once, highlighting the generality of this approach. The interested reader is also referred to the Supplementary Materials Section S1 for a practical example of how to use this scheme to derive all formation energies starting from a given descriptor pair.

The derived multi-linear relations Δ_*i*_*E*_0_(*U,pH*; Δ_CO_*E*_0_, *U*^PZC^) and Δ_*i*_*a*(*U,pH*; Δ_CO_*E*_0_, *U*^PZC^) given in Supplementary Materials Table S2 fully define the thermodynamic free energy landscape at a given pair of descriptor values. To also define kinetic barriers, the approach of Hansen et al. is adapted which first fixes the activation barrier at the equilibrium, reversible potential of each elementary step to a defined value^36^. This idea is extended to include the effect of surface charging on the reversible potential following Eqs. (2a) and (2b). Potential dependence is then included via the BEP approach for which previous theoretical studies have indicated the validity^21^. The resulting linear scaling of the change of the thermodynamic driving force Δ_*i*_Ω across elementary steps using a charge transfer coefficient *β*_*i*_ leads then to a Butler-Volmer-like equation (see Methods section B for details). This gives rise to two more parameters for the two electrochemical barriers, the fixed barrier at the reversible potential ${G}_{{{{{{{{\rm{a}}}}}}}},{{{{{{{\rm{i}}}}}}}}}^{\circ }({U}_{i}^{{{{{{{{\rm{rev}}}}}}}},\circ })$ and the parameter *β*_*i*_ defining the initial/final state-likeliness (both in terms of energetics and field-dependence) of the transition state and thus also its potential dependence. Instead of calculating these two parameters, I rationalize their values from previous experimental studies^30^. The reader is referred to Methods section B for a detailed discussion of this procedure and also the reasons for neglecting additional kinetic barriers for CO desorption and CO_2_ adsorption.

### CO activity volcano

**Fig. 2:**
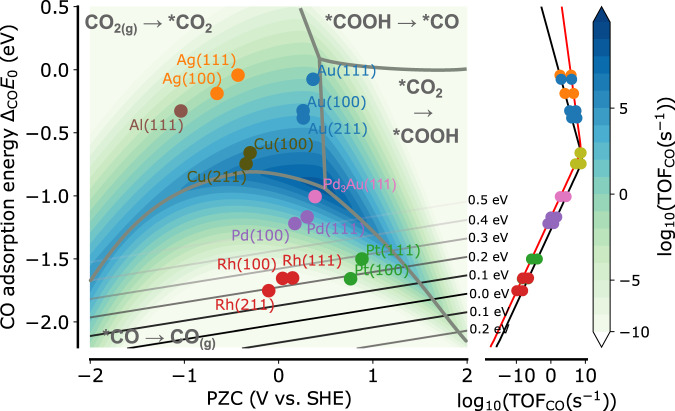
Charge-corrected descriptor-based volcano for the CO_2_RR to CO activity at -1 V vs. SHE (pH 6.8). Contour lines depict the turnover frequency for CO_2_ to CO conversion from the descriptor-based kinetic model as a function of PZC and CO adsorption energy (relative to CO gas and without ZPE, free energy and gas corrections). Solid contour lines represent iso-lines of the absolute value of the hydrogen adsorption free energy, predicted as a function of the two descriptors. The right panel shows the conventional CO_2_RR to CO activity volcano which does not consider the CO_2_ intermediate or surface charging. It was generated using the kinetic parameters in this work (black) and the original kinetic model (red)^36^ taking adsorption energies at the potential of zero charge to be correlated with the CO adsorption energy only. Source data are provided as a Source Data file.

Starting from activation energies of all elementary steps, a mean-field micro-kinetic model (MKM) for the reaction mechanism presented in Eq. (1) is constructed and solved in the steady-state approximation using the CatMAP program package^11^. Fig. 2 shows the activity volcano plot of the turnover frequency (TOF) of CO formation obtained from the MKM model as a function of the two descriptors, CO adsorption energy, and PZC, at −1.0 V vs. SHE, the voltage at which CO is primarily formed on most CO forming metals^29,33^. In the following, this two-dimensional model co2-2d is compared to the conventional CO_2_RR to CO kinetic model (co2-1d)^36^ which does not consider the presence of the *CO_2_ intermediate nor the presence of surface charging.

By means of a degree of rate control analysis, it is first found that the CO activity volcano in the co2-2d model decomposes into four distinct regions with different rate-limiting steps (shown in Fig. 2). The co2-1d model becomes instead a one-dimensional activity volcano as a function of the *CO adsorption energy^36^ and shows only two different rate-limiting steps, *COOH to *CO for the weakly CO-binding, and *CO desorption for the strongly CO-binding leg. For very weakly CO-binding materials, the co2-2d model shows that the *COOH to *CO step is only rate-limiting for materials with a positive PZC, and thus those which have significant surface charge density at −1 V vs. SHE (cf. Eq. (2b)), stabilizing the adsorption of *CO_2_. At more negative PZCs, however, CO_2_ adsorption is always rate-limiting. This can be explained by the stronger adsorption dipole of *CO_2_ which results in at least one order of magnitude larger Δ_*i*_*a* (cf. ref. ^29^ and Supplementary Materials Table S1) that destabilizes *CO_2_ significantly more compared to *COOH when moving to more negative PZC values, and thus reduced negative surface charge density.

At highly negatively charged electrodes with a very positive PZC, another transition can be found at intermediate CO binding strengths for which the *CO_2_ to *COOH step becomes limiting. As seen in Supplementary Materials Table S2, both *CO_2_ and *COOH adsorption energies have a similar *CO adsorption energy scaling of around ≈0.6 which makes them become less stabilized compared to *CO when going to more strongly *CO binding metals. Thus, the *COOH to *CO step becomes increasingly likely while the previous step becomes rate-limiting. As expected, the *COOH to *CO region enlarges in both PZC and CO adsorption energy direction when increasing the reversible potential activation barrier of the *COOH to *CO step (Supplementary Materials Fig. S6). As explained in the Methods section B, this barrier is chosen based on experimental results on Au, and affects only the rate-limiting step regions, not the actual value of the TOF (Supplementary Materials Fig. S6).

Since the *CO intermediate is the one that is most strongly stabilized when moving to strongly CO-binding metals, CO desorption becomes rate-limiting inevitably (Supplementary Materials Table S2). It is notable though that the transition line marking this region has a strong curvature resulting from the PZC dependence of the CO activity in the co2-2d model. This distinguishes the volcano substantially from the co2-1d model. The curvature of the volcano indicates that catalysts with the same CO adsorption energy can exhibit significantly different activity and thus also free energy diagrams depending on their PZC. As explained above, *CO_2_ is significantly more sensitive to the PZC compared to the other intermediates, being strongly stabilized when going to more positive PZC values (cf. Table S2). Therefore, at a fixed *CO adsorption energy and relatively strong *CO binding, an increasing PZC will at some point always make the *CO_2_ to *COOH step rate-limiting. On the other hand, for a very negative PZC, CO_2_ is drastically destabilized which will make CO_2_ adsorption rate-limiting at some point. The result is a defined PZC region in which *CO desorption is rate-limiting which includes all the transition metals that were investigated in this work.

All metals adsorbing CO weaker or equal to Cu lie in the region where CO_2_ adsorption is rate-limiting. In this region (with the exception of Cu), a negligible coverage of all reaction intermediates is found (cf. Supplementary Materials Fig. S8) in line with experimental works on Au^57^. If CO_2_ adsorption is rate-limiting, no pH-dependence is expected of the CO partial current on an absolute potential scale, even under acidic conditions, since no proton is involved in the rate-limiting step and all coverages are small so that the pH-dependence of the number of free active sites is negligible. This prediction from our model has been indeed recently verified on Au^30^, Ag, Sn and In^41^. Previous experimental studies have shown that at −0.7 V vs. SHE, the pH-dependent formation of *COOH becomes rate-limiting on Au^30^. Since I used this experimental result to parametrize the kinetic model (cf. Methods section B), our model reproduces this transition quantitatively, and the rate-limiting step region of *COOH to *CO moves to more negative PZCs so that it is located exactly over Au at this potential (cf. Supplementary Materials Fig. S10). More importantly, however, the co2-2d model can also reproduce that on Ag, a corresponding switch of the rate-limiting step associated with a reduction of the Tafel slope from 120–140 mV/dec^33,58^ to around 80 mV/dec^58^ is observed already at around −0.4 V vs. SHE (Supplementary Materials Fig. S10). Our model reveals that the physical reason for this earlier switch to CO_2_ adsorption being limiting is the less positive PZC of Ag reducing the interfacial electric field and thus the driving force for CO_2_ adsorption. The agreement with these trends increases the trust in the transferability of our kinetic model and the developed descriptor space.

An additional indication that our model is an improvement to the conventional model is the comparison between Au and Ag. The right panel of Fig. 2 shows the conventional activity volcano constructed from the CO adsorption energy as the mere descriptor without any account for the *CO_2_ intermediate nor for surface charging (adsorption energies from Δ_*i*_*E*_0_). Kinetic parameters are either taken to be the same as in the two-dimensional volcano (black line) or from the original published model (red line)^36^. By taking the (100) facet as an example, the CO TOF increases by 0.7 orders of magnitude for the original co2-1d model, and 1.4 orders of magnitude for the updated co2-1d model. From analyzing previous experimental data in Supplementary Materials Fig. S7^30,33,59,60^ on various planar Au and Ag surfaces in H-cells, it becomes, however, clear that for the same facet, Au surfaces are commonly up to three orders of magnitude more active than Ag in the lower kinetic potential region (which is not limited by mass transport^30^). By comparing to Fig. 2, the co2-2d model captures this order of magnitude and shows that the difference to the co2-1d model arises from the non-horizontal TOF contour lines in the region where CO_2_ adsorption is rate-limiting. These arise due to the CO_2_ intermediate which is increasingly stabilized when moving to more positive PZC values due to the increased negative surface charge density explaining the much higher activity of Au compared to Ag.

The fact that CO_2_ adsorption is rate-limiting on all strongly CO-producing metals suggests that rather than the pH, the surface charge density might be a much more effective parameter to tune the CO production rate (cf. also Fig. S9 showing a minor pH effect). In addition, since Au under-binds CO, the addition of a strongly CO-binding metal like Pd might be moving the system into the more active region, as recently experimentally demonstrated for Au-Pd nanoparticle catalysts^61^ and depicted here by the Pd_3_Au alloy catalyst in Fig. 2. Another interesting observation is that Al(111) is predicted to be reasonably active. Although thermodynamically, Al is likely to be at least partially oxidized under electrochemical CO_2_RR conditions^62^, the co2-2d model suggests that the remaining Al sites might be very active and indeed Al-based bimetallics have been already reported as promising catalysts ^24^.

For all considered strongly CO-adsorbing metals, CO desorption is rate-limiting, and the surface is fully covered with CO (cf. Supplementary Materials Fig. S8). According to the co2-2d model, Pd is a catalyst with impressively high activity comparable to Ag. Pd is indeed also experimentally known to produce CO^63–65^, albeit it is rarely discussed as a promising CO_2_RR catalyst due to the prevailing HER. To visualize the regions of high HER rate, the hydrogen adsorption energy (without accounting for the field interaction which is a small effect^32^) is correlated with the two descriptors revealing a high *R*^2^ = 0.90. This correlation originates mostly from the correlation with the CO adsorption energy (*R*^2^ = 0.87), leading to almost horizontal isosurfaces of the H adsorption energy in Fig. 2, with the stronger binding CO metals showing increased HER activity. The high CO coverage, however, can to some degree suppress HER by poisoning active sites^66^. Therefore if means to suppress HER can be employed and improved, such electrocatalysts might become highly promising candidates^67,68^. Finally, I also mention that due to saturation of the CO coverage (cf. Supplementary Materials Fig. S8), the CO formation rate is independent of pH also in this region, suggesting again a minor role of the pH in enhancing the CO production rate (cf. also Supplementary Materials Fig. S9 showing the pH effect on the activity volcano).

The most interesting part of the activity volcano is the central high CO activity region, where the rate-limiting step switches from CO_2_ adsorption to CO desorption. Cu lies according to our model closest to this region with the binding of CO being neither too weak nor too strong leading to a partially CO-covered surface (Supplementary Materials Fig. S8 and ref. ^69^). The three to four orders of magnitude higher activity of Cu vs. Au stands in contrast to the previously published model^36^ (shown in the right panel of Fig. 2, red line) suggesting Au has comparable or even higher TOF (looking at the (211) facet). Using our kinetic parameters, however, the co2-1d model gives a comparable order of magnitude change from Au to Cu (right panel in Fig. 2, black line). Both co2-1d models, however, locate the maximum of the activity volcano at a too-weak CO binding, as well as not give an account for the strong curvature with PZC. The latter is important, as it can easily move a catalyst outside the active region, despite a perfectly matching CO adsorption energy. Experimentally, further reduced products like C_1_ (methane) and C_2_ (ethylene) products are usually observed on Cu, depending on the potential region and pH^70,71^. Our calculations suggest, however, that Cu is actually a very excellent catalyst for producing CO, indicating that a rather significant amount of higher reduced products might be formed by re-adsorption of CO. Also the combination with a higher PZC and stronger CO binding metal like Pd might be promising as it moves the system into the higher activity region, and corresponding alloys showing high CO selectivity have been recently reported^72^.

### Tuning bimetallic alloys for CO production

**Fig. 3:**
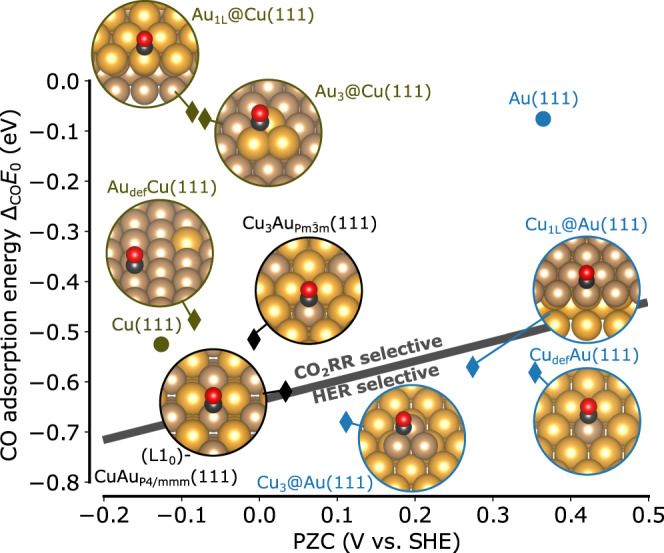
Effect of Cu-Au bimetallic composition and structure in the here reported descriptor space. The gray line indicates a transition from CO_2_RR to HER selectivity. Circles refer to the data points which were actually used for the training our descriptor model, and diamonds for those where only the descriptor values were evaluated. Source data are provided as a Source Data file.

The maximum of the co2-2d volcano lies in a region of space where no bare transition metal surface is located. In principle, transition metals might be combined for example in the form of alloy compounds, resulting in hybrid properties between the two materials making it possible to navigate through the descriptor space. Multi-metallic catalysts could be formed by doping a substrate with a different metal e.g. in the form of single atoms^73^, forming an overlayer^74^, forming homogeneously mixed high entropy alloys^75^, pure alloy crystal phases, nano-particles, etc^76^. In Fig. 3 these possibilities are evaluated at the example of Au-Cu bimetallic alloys to discuss how such structural motifs could help to navigate in the here reported descriptor space. CO will always adsorb on the most strongly binding site and thus the presence of Cu sites on the surface will always set the CO adsorption energy descriptor to a value close to Cu. Variations of this adsorption energy across the bimetallics are rather small, only about ± 0.1 eV. This suggests that the tuning of material properties based on adsorption energies may not be particularly successful in actually designing better catalysts. I stress that this simplified treatment does not take into account coverage distributions across active sites, a saturation of the Cu sites, etc. However, in contrast to the comparably difficult modulation of the CO adsorption energy, the PZC can be much more easily tuned over a wide range. Looking at this descriptor in more detail, one can first note by comparing analogous surface configurations that the less noble metal dominates the PZC. In order to increase the PZC of Cu to make it more active for CO formation, the most efficient way is to dope Cu into an Au substrate. Also having small Cu clusters on an Au substrate can be beneficial up to a full monolayer of Cu. In contrast, high entropy alloy or single crystalline crystals promise much less tunability of the PZC, fixing the value to the less noble metal. The importance of the substrate in tuning the PZC of a catalytically active site on the surface has been so far relatively rarely discussed^35^ but could indeed play a much more important role than previously expected. By comparing to Fig. 2, increases of the PZC relative to Cu would activate the catalyst for the formation of CO. CO producing Cu-Au alloy catalysts where Cu and Au are mixed homogeneously in the surface have been reported in the literature ^77^.

### Description of experimental product selectivity trends

So far, the introduced two-dimensional descriptor space was mainly discussed based on the CO_2_ to CO conversion rate landscape. However, a large part of the metals presented in the figures so far is not actually selectively forming CO during CO_2_RR, but instead formate/formic acid, C_1_- or C_2_- products or even performing the HER. The construction of a kinetic model for all of these processes has been recently attempted including suggestions of descriptors like the *CO, *OH, *C and *H adsorption energies^37,38,78,79^, but these models still lack in their ability to explain all selectivity trends^78^. An interesting outcome from previous studies was the discovered correlation of the adsorption energies of all intermediates in the CO_2_RR process on Cu to C_1_- or C_2_- products with the CO adsorption energy^80^. This suggests that the same descriptor pair that I suggest here might be again valid also to describe the activity for other products and thus also the selectivity trends. In addition, the formation of C_2_ products is limited by the field-driven coupling of two *CO intermediates^28,29,69,81–87^ which could suggest the necessity of the PZC descriptor to explain selectivity trends.

**Fig. 4:**
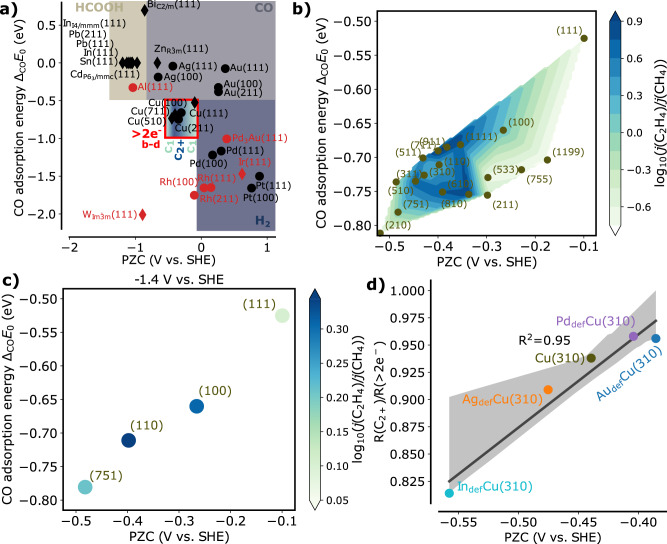
CO_2_RR product selectivity as a function of the here-developed descriptors. **a** Main experimental product selectivity^30,33,59,64,90,134–137^ for electrochemical CO_2_RR as a function of the developed descriptor space. Red markers: No selectivity data available. The red-boxed Cu region is discussed in detail in (**b** and **c**). **b** Ethylene/methane selectivity on a log scale as a function of the here-developed descriptors. The data was taken from constant-current experiments by Hori et al^90^. and interpolated via a triangular, linear fit. **c** Ethylene/methane selectivity on a log scale as a function of the here-developed descriptors. The data is obtained from extrapolating constant potential data^83,90,138^ to the same potential of −1.4 V vs. SHE. **d** Product selectivity for C_2+_ products vs. products beyond formate and CO ( > 2e^−^) as extracted from ref. ^91^ at −1.3 V vs. SHE plotted vs. the PZC. Source data are provided as a Source Data file.

If the discovered descriptors would be useful also for the description of other product pathways, a plot of the experimentally revealed dominant product selectivity as a function of these descriptors should clearly separate into selectivity regions^88^. Figure 4a shows the experimentally determined dominant product of electrochemical CO_2_RR at −1.4 V vs. SHE and pH = 6.8 from studies using planar electrodes (details in Supplementary Materials Section S3). As seen from this picture, the descriptor space clearly separates into defined regions of specific product selectivity. While a large part of the descriptor map is relatively unexplored, such as for example Rh, this suggests that the descriptors may also be suitable to screen catalysts for a desired product of choice. Particularly notable is also again the different impact of the two descriptors. The CO adsorption energy distinguishes the CO_2_RR- from the HER-selective region. This can be understood by the fact that stronger CO binding surfaces are generally poisoned by *CO^66^, but in turn have an almost optimal *H adsorption energy (cf. the H-adsorption energy lines in Fig. 2). CO_2_RR activity is only expected if the CO adsorption energy is in the intermediate to weak region (< −1 eV) where *H adsorption is also significantly weaker so that CO_2_RR becomes competitive. Further reduced products compared to formate and CO are only formed if *CO can be more stabilized on the surface so that it can further react, as in the case of intermediately strongly CO-binding metals such as Cu. The PZC determines the product selectivity of CO_2_RR. At weaker CO binding, the PZC distinguishes formate vs. CO selectivity. An interesting fact, since it has been noted before^35^ that CO formation might be a field-sensitive process, driven at positive PZC values, while formate is produced via CO_2_ reacting directly with *H^89^, a field insensitive process^35^. The latter becomes the dominating path if the CO_2_ adsorption step is very uphill in energy, which is the case at more negative PZC values, blocking the CO-forming pathway. This might explain why the PZC is effective in distinguishing formate from CO selectivity.

To gain more insights into the region of higher reduced products, meaning the formation of methane, ethylene, ethanol, etc., the Cu region is enlarged, and experimental data is analyzed in detail using the here-developed descriptors. Hori et al. recorded the ethylene vs. methane selectivity at a constant current on a variety of Cu single crystalline facets^90^, and this is shown in Fig. 4 b. The data shows an interesting volcano-like trend with a maximum selectivity for ethylene at a PZC of −0.4 V vs. SHE. When looking at these trends, it is also important to consider typical experimental error bars, which can change the selectivity easily by 0.1^30^. Therefore, the selectivity-descriptor relation is refit by a Gaussian process regression with regularization accounting for systematic noise. As Supplementary Materials Fig. S11 shows, this treatment smooths out the variations in the direction of the CO adsorption energy resulting in a simple one-dimensional selectivity volcano as a function of the PZC. Unfortunately, the data of Hori et al. was recorded at constant current, and the selectivity drastically changes with potential^37^. To explore this point in more detail, potential-dependent CO_2_RR data on Cu single crystals is extracted from literature and plotted in the same figure (Supplementary Materials Fig. S12). The data is then interpolated to estimate the selectivity at a constant potential (Supplementary Materials Fig. S13). From this much sparser data set, a similar dependence on PZC can be found, as seen in Fig. 4c with a maximum at a similar position for −1.4 V vs. SHE which, however, shifts with potential (Supplementary Materials Fig. S14). While the sparsity of the experimental data makes it hard to make final conclusions about the location of the selectivity maximum, it is interesting to see, how well the two descriptors can define the selectivity space, once the relevant experimental data is present.

To explore the generality of the volcano-like PZC trend of the ethylene/methane selectivity, the PZC descriptor is also applied to predict the C_2_ vs. > 2e^−^ reduction (including all products but CO and formate) selectivity on metal-doped Cu catalysts from recent experimental data^91^(Fig. 4b). Interestingly, the descriptor correlates nicely with the selectivity data with the exception of the experimental data on Sn which has been also noted as a special case in the original paper^91^. The enhancement of C_2_ product selectivity with increased PZC can be explained by increased surface charge density (Eq. (2b)) and the resulting increased electric field. It has been previously shown that the coupling of two *CO intermediates that is rate-limiting for C_2_ product formation has a strong field-dependence and correspondingly large Δ_*i*_*a*-coefficients^28,29,69,81–86^. Due to this, a more positive PZC usually increases the C_2_ over C_1_ product selectivity. This is in line with the low-PZC branch of the selectivity volcano predicted from analyzing the experimental Cu facets data in Figs. 4b and c. This also agrees with recent experimental data on organic molecule-functionalized Cu surfaces showing higher surface charge density to enhance the C_2_ product selectivity^92^. At even higher PZC values, the selectivity is predicted by this analysis to go down at some point, likely due to an over-stabilization of *OCCO and the next step becoming rate-limiting.

I also compared our descriptor space to the one more conventionally discussed in the community consisting of CO and H adsorption energies^88^. Supplementary Materials Fig. S15 shows the selectivity map which reveals important differences to our map. First, as I discussed above, the H adsorption energy correlates much more closely with the CO adsorption energy than the PZC leading to selectivity separation lines lying on the diagonal instead of vertical and horizontal. Secondly, the H adsorption energy cannot efficiently separate the different product selectivity regions, like formate and CO, or the trend from C_1_ to C_2_ products. Another problem with using this descriptor is the assignment of the correct adsorption site for H. In addition to the general problem of assigning an active site, some arbitrariness arises for example in the question if this should be the most stable site on a chosen facet or the CO_2_RR active site. These findings indicate, that the PZC – which depends on the facet, but is roughly independent of local variations in the field, and thus particular active site environments – might be a more useful addition to the CO adsorption energy descriptor to rationalize experimental selectivity trends.

## Discussion

The results of this work highlight the importance of the PZC as an electrochemical design parameter. It is noted that this study is not the first to notice the significance of the PZC. In particular, in the HER community, activity-work function and activity-PZC trends have been discovered^93–95^, but until today not fully understood. Possible explanations have been suggested, e.g. that a more positive PZC leads to a more negatively charged electrode and thus enthalpic stabilization of the charged transition state which is partially compensated by entropic changes due to proton accumulation^94,96^. Here, I presented a fully enthalpic explanation. While the adsorbed *H is charge-neutral at first sight, it still transfers charge and thus exhibits a small^32^, but significant^97^ interfacial dipole which can be stabilized by e.g. going to more positive PZC values. Without making any claims on the possibly significant entropic contribution, this work suggests a fundamental, enthalpic explanation for the work function or PZC dependence of electrochemical processes and demonstrated the high significance, especially for electrochemical CO_2_ reduction.

To prepare materials with optimal PZC, structural design concepts need to be developed. Such concepts are relatively well known for the work function: Halide adsorption^98^ or the adsorption of organic molecules^92,99^ are just a few examples, of how the work function can be tuned in addition to the substrate effect presented above. Employing work function design strategies the future task should thus be to find materials with the optimal CO adsorption energy and PZC. For this, high-throughput computational screening of materials should be performed which can be facilitated by machine learning methods that utilize geometric and physical features. The prediction of the adsorption energy is thereby tricky, as it requires knowledge about the active site which is not always obvious from experimental setups. Besides that, the development of material features for learning CO adsorption energies^100^ or work functions^101,102^ is in process and can be utilized in this plan.

Moreover, I comment on the PZC that I used throughout this manuscript and its suitability as a descriptor for high-throughput material screening. The PZC that I used is ultimately an ideal, theoretical value that is comparable roughly to the experimental PZC in the absence of specific adsorption and reconstruction (Supplementary Materials Fig. S1). It can be theoretically directly obtained from the DFT calculation of the slab system from the Fermi level and the relation presented in Supplementary Materials Fig. S1 (practically, a new relationship might need to be obtained to reference vs. true vacuum). Experimentally, the PZC, or as it is also called the potential of zero free charge (PZFC) is prone to additional environmental effects such as the type of anions or cations, or the pH due to specific adsorption and reconstruction^27,103^ and is generally measured with a larger uncertainty, although recently it has been even assigned to grains of a catalyst surface^93^. Therefore, for comparison of the presented theory with experimental systems, it might be also useful to make use of the advances of vacuum techniques to measure the more accurately measurable work function^55^ instead and use the relation with the PZC^50^, such as that presented in Supplementary Materials Fig. S1 to derive the theoretical PZC that is used for all plots presented above. I stress that it is the ideal, theoretical PZC or work function that explains the selectivity trends e.g. in Fig. 4, rather than the true PZC. Of course, for more complex systems which are prone to reconstruction or specific adsorption, the model should be revised again using theoretical methods to resolve the double layer in atomic detail.

Lastly, I also comment on the importance of cation effects for the activity and selectivity trends presented here. Cation concentration and identity have been shown to affect both HER^104–106^ and CO_2_RR^29,30,32,35,106–110^ conversion efficiency and product selectivity. For CO_2_ reduction, cations affect the selectivity mostly be stabilizing key intermediates by direct bonding and field stabilization^29,107,108^. Thus, our presented activity trends could shift substantially under different electrolyte environments, a point which should be explored in future studies. According to our previous work, mono-valent cation effects could be incorporated by changing the surface charging relation used in this work to one including cation-size effects^29^.

In this work, I introduced the PZC as a so-far underrated parameter to describe activity and selectivity trends in electrochemical energy conversion. The descriptor arises from the electric double layer field which influences the stability of certain reaction intermediates which give rise to a strong interfacial dipole, such as e.g. the *CO_2_ intermediate in the electrochemical reduction of CO_2_. To show this, I performed DFT calculations on metallic catalysts accounting for the electric double layer and found that the full free energy diagram can be mapped onto a two-dimensional descriptor space composed of CO adsorption energy and PZC. From micro-kinetic modeling, I found the PZC to effectively break the scaling of intermediate formation energies opening up new material design spaces. Due to the global nature of the PZC, this design space is much more flexibly accessible via the PZC compared to adsorption energies. Using the here-developed descriptor space, I explained a variety of experimentally observed trends in CO_2_ reduction, shedding much trust in the model. An important outcome is for example a volcano-like dependence of the C_2_ vs. C_1_ product formation rate on the PZC. Our results indicate that the PZC represents a so far less recognized, but important parameter for the discovery of electrocatalysts and the explanation of experimental trends and findings.

## Methods

### A: DFT calculations

#### Conceptual details

The adsorption energy (or grand potential^26,44^) of any reaction intermediate *i* is defined as:

4 $${\Delta }_{i}\Omega (U)=	{G}_{i}(U)-{G}_{*}(U)-{N}_{{{{{{{{\rm{C}}}}}}}}}{\mu }_{{{{{{{{\rm{C}}}}}}}}}\\ 	-{N}_{{{{{{{{{\rm{H}}}}}}}}}^{+}+{{{{{{{{\rm{e}}}}}}}}}^{-}}({\tilde{\mu }}_{{{{{{{{{\rm{H}}}}}}}}}^{+}}+{\tilde{\mu }}_{{{{{{{{{\rm{e}}}}}}}}}^{-}})-{N}_{{{{{{{{\rm{O}}}}}}}}}{\mu }_{{{{{{{{\rm{O}}}}}}}}},$$

with *U* denoting the electrochemical potential of electrons in the electrode at which the energies of the adsorbed and empty slab state are evaluated vs. the SHE reference electrode, i.e. the electrode potential on a SHE scale. *N*_*i*_ denotes the number of each adsorbate element, where the elemental reference chemical potentials *μ* are given as:

5 $${\tilde{\mu }}_{{{{{{{{{\rm{H}}}}}}}}}^{+}}+{\tilde{\mu }}_{{{{{{{{{\rm{e}}}}}}}}}^{-}}	={\mu }_{{{{{{{{{\rm{H}}}}}}}}}_{2}}-eU-0.0592\,\,{{{{{{{\rm{eV}}}}}}}}\cdot pH\\ {\mu }_{{{{{{{{\rm{O}}}}}}}}}	={\mu }_{{{{{{{{{\rm{H}}}}}}}}}_{2}{{{{{{{\rm{O}}}}}}}}}-2({\tilde{\mu }}_{{{{{{{{{\rm{H}}}}}}}}}^{+}}+{\tilde{\mu }}_{{{{{{{{{\rm{e}}}}}}}}}^{-}})\\ {\mu }_{{{{{{{{\rm{C}}}}}}}},{{{{{{{{\rm{CO}}}}}}}}}_{2}}	={\mu }_{{{{{{{{{\rm{CO}}}}}}}}}_{2}}-2{\mu }_{{{{{{{{\rm{O}}}}}}}}}\\ {\mu }_{{{{{{{{\rm{C}}}}}}}},{{{{{{{\rm{CO}}}}}}}}}	={\mu }_{{{{{{{{\rm{CO}}}}}}}}}-{\mu }_{{{{{{{{\rm{O}}}}}}}}}.$$

In the microkinetic modeling framework, we always use the CO_2_(g) reference, but in the plots shown in the main paper, the CO reference is used to define the adsorption energy of CO. The electrochemical potentials of protons and electrons are defined via the CHE approach which assumes electrochemical equilibrium at the SHE reference electrode allowing for re-reference of the electrochemical potentials^19,20^. We further defined for convenience:

6 $${\Delta }_{i}\Omega (U)={\Delta }_{i}{E}^{U}(U)+{\Delta }_{i}{E}^{{{{{{{{\rm{T}}}}}}}},{{{{{{{\rm{S}}}}}}}},{{{{{{{\rm{ZPE}}}}}}}},\circ }+eU+0.0592\,\,{{{{{{{\rm{eV}}}}}}}}\cdot pH,$$

where we integrated the DFT-calculated properties into a single term Δ_*i*_*E*^*U*^. The corrections for finite pressure (standard conditions) and temperature and zero-point energy were moved into the term Δ_*i*_*E*^T,S,ZPE,∘^. To obtain Δ_*i*_*E*^*U*^, a standard approach is to utilize charge equilibration to match the Fermi levels (= electron’s electrochemical potentials) of the initial and final state (grand-canonical constant potential approach) and then obtain the potential-dependence by varying the excess charge^44,111^. Here, we disregard the often small charge equilibration contribution and approximate the energy difference as a constant charge energy difference Δ_*i*_*E*^*σ*^^30,44,111^, for simplicity here denoted as Δ_*i*_*E*:

7 $${\Delta }_{i}{E}^{U}(U)\,\approx \,{\Delta }_{i}E(\sigma )$$

With that, the adsorption energy becomes:

8 $${\Delta }_{i}\Omega (U)={\Delta }_{i}E(\sigma (U))+{\Delta }_{i}{E}^{{{{{{{{\rm{T}}}}}}}},{{{{{{{\rm{S}}}}}}}},{{{{{{{\rm{ZPE}}}}}}}},\circ }+eU+0.0592\,\,{{{{{{{\rm{eV}}}}}}}}\cdot pH,$$

The surface charge density is related to the potential by a simple plate-capacitor equation:

9 $$\sigma={C}_{{{{{{{{\rm{gap}}}}}}}}}\cdot (U-{U}^{{{{{{{{\rm{PZC}}}}}}}}}),$$

with the Helmholtz or gap capacitance *C*_gap_ and the potential of zero charge (PZC) vs. SHE *U*^PZC^. This is valid because, under electrochemical conditions, the diffuse layer contributions to the surface charge density are small^29^. The PZC is accessible from the simulations via the Fermi level *ϵ*_*F*_. In our simulations, we use a small dielectric permittivity of *ε*^b^ = 6 (see computational details discussion) defining the potential reference. The Fermi level or work function is in our system thus measured relative to almost vacuum (*ε*^b^ = 6). We found a fairly linear correlation of the experimental PZC vs. SHE and the theoretical PZC − *ϵ*_*F*_/*e* vs. *ε*^b^ = 6 from a series of metallic facets (Supplementary Materials Fig. S1), related merely by a constant shift of 4.92 V:

10 $${U}^{{{{{{{{\rm{PZC}}}}}}}}}=-{\epsilon }_{F}/e-4.92V.$$

The constant shift represents the absolute SHE potential within the implicit solvent model applied^26^. For the gap capacitance we took the approximately metal-independent value of around 20 μF/cm^2^^47–49^. The advantage of this formulation compared to the constant potential formulation is that it avoids the problem of implicit solvation models not accurately producing the capacitance value across several metals using a single implicit solvation model parameter set^112^. We note that the real picture of the electric double layer might be more complex than described here, with specific adsorption, effects of the diffuse layer, ionic correlations, etc. but in many cases, even a simple plate-capacitor motivated model can correctly describe trends across materials^26^. This is also since the capacitive response of most metal electrodes under highly polarizing CO_2_RR conditions of −1 to −1.5 V vs. SHE is dominated by the Helmholtz response with only small contributions of the diffuse layer^26,29^.

To evaluate Δ_*i*_*E*(*σ*), we employed an implicit solvent model and a planar counter charge representation of the mobile counter-charges. From this, we found an approximately linear dependence, so that:

11 $${\Delta }_{i}{E}^{U}(U)\,\approx \,{\Delta }_{i}E(\sigma (U))\,\approx \,{\Delta }_{i}{E}_{0}+{\Delta }_{i}a\cdot \sigma .$$

We note that this approximation will generally break if the capacitance changes during the adsorption event (the 2nd-order term). In our case, we found this term, however, to be relatively minor and similar to previous studies^43,45^. The charge sensitivity parameters Δ_*i*_*a* are related to the change of the PZC during the adsorption event, or in other words, the partial charge transferred during the adsorption event, or electrosorption valence^26,43,44^. The partial charge transferred generates an electric dipole moment, which modifies the PZC and interacts with the interfacial electric field. In summary, in our approach, the potential-dependent free energy diagram is completely defined from three formation energies Δ_*i*_*E*_0_, the field sensitivity parameters Δ_*i*_*a*, the PZC of each material *U*^PZC^ and the finite temperature and zero-point energy correction term Δ_*i*_*E*^T,S,ZPE,∘^.

#### Technical details

Density functional theory calculations of reaction energetics were carried out with a periodic plane-wave implementation and ultrasoft pseudopotentials using QUANTUM ESPRESSO version 6.1^113^ interfaced with the Atomistic Simulation Environment (ASE)^114^. We applied ultra-soft pseudopotentials and the BEEF-vdW functional, which provides a reasonable description of van der Waals forces while maintaining an accurate prediction of chemisorption energies^115^. The bulk lattice constant of the face-centered cubic (fcc) materials was optimized within QUANTUM ESPRESSO using spin-polarized DFT, plane-wave, and density cutoffs of 900 and 9000 eV, respectively, 1 × 1 unit cells, and 13 × 13 × 13 Monkhorst-Pack k-point grids^116^, until forces were converged to less than 0.05 eV/Å.

Following the bulk relaxation, surfaces were cut using ASE^114^, CatKIT^117^ and Pymatgen^118^. For the face-centered cubic (fcc) (111) and (100) surfaces, large 4 × 4 orthogonal supercells were constructed to minimize adsorbate-adsorbate interactions and approach the limit where constant charge and constant potential calculations approach each other^43^ and a 3 × 3 × 1 Monkhorst-Pack k-point grid used^119^, which led to converged energies and Fermi level. Correspondingly, orthogonal 3 × 4 supercells and 4 × 3 × 1 Monkhorst-Pack k-point grids were used for the fcc(211) surfaces. For all other surfaces, the separation of surface adsorbates was attempted to be chosen similarly to the other surfaces and the k-point grid density was kept as similar as possible. Adsorption energies were evaluated using asymmetric (adsorbates on one side of the slab) three-layered supercells with the lowest layer constrained and 20 Å separation of the surface slabs. Spin-unpolarized calculations were performed using plane-wave and density cutoffs of 700 and 7000 eV, respectively, as well as a Fermi-level smearing width of 0.1 eV. The reported Fermi energies in the figures have been, however, mostly obtained using lower plane-wave and density cutoffs of 500 and 5000 eV, respectively.

The SCCS implicit solvation model as implemented in the Environ QUANTUM ESPRESSO module^120^ was used to model the presence of implicit water in all surface slab calculations. We employed the “fitg03” (in a.u.: ${\rho }^{\min }=0.0001,{\rho }^{\max }=0.005$) solvation parameter set which has been optimized for neutral molecule solvation energies^120^. The bulk relative dielectric permittivity was set to *ε*^b^ = 6 corresponding to highly constrained water that has been rationalized in our previous study^29^. Cavitation and repulsive energy terms are included by introducing an energy term proportional to the cavity surface area as described in ref. ^120^ and we here apply the parameter (*α* + *γ*) = 11.5 dyn/cm from the fitg03 parameter set. Dispersion interactions are ignored since they depend on the cavity volume which is an ill-defined property in surface slab calculations. The surface charge density was modulated in order to simulate the response of adsorbate-free energies to the presence of an electric double-layer field. A planar counter charge with a slab separation of 5 Å was applied to neutralize the simulation cell. A parabolic correction was applied in the Environ calculations to decouple the electrostatic interaction between the periodically repeated slabs.

All structures were relaxed at the corresponding surface charge density using a BFGS line search algorithm until force components were less than 0.05 eV/Å. *CO_2_ requires for many metallic surfaces the presence of negative excess surface charge to be stable. In line with our previous work^29,30^, we, therefore, extrapolated the formation energy for smaller surface charge densities by performing single point calculations using the optimized geometry at the least negative surface charge where the adsorbate still adsorbed. In general, the most stable adsorption sites were searched for by testing various adsorption sites, despite the (111) facets, where the most stable sites are well known (fcc or hcp hollow for ^*^H, top for ^*^CO, bridge bonded configuration for ^*^COOH, see e.g.^33^).

Zero-point energy and finite temperature corrections in the harmonic oscillator approximation were taken from our previous work on gold^30^, and it was assumed that these values do not change much between different metals and facets. We applied an energy correction of 0.33 eV to the gas phase energy calculation of CO_2_, which was determined from experimental gas-phase reaction energies^115^. This results in an equilibrium potential for CO_2_ reduction to CO of −0.04 V vs. SHE (pH = 0) which is close to the experimental value of −0.10 V vs. SHE^70^. Further, double bond corrections of +0.25 eV were applied to *COOH and *CO_2_^69^, and a hydrogen bonding solvation correction of −0.25 eV to the energy of *COOH^121^, due to the underestimation of this contribution by implicit solvation techniques^122^. The corrections were the same as in ref. ^30^.

### B: Micro-kinetic modeling

#### Conceptual details

Micro-kinetic modeling was performed using the CatMAP package^11^. CatMAP solves the mean-field rate equations (all implemented as reversible) in the steady-state assumption. Within CatMAP, Butler-Volmer kinetics^123^ for proton-coupled electron transfers (PCETs) are implemented for each elementary step^36^. We extended this formulation to also include surface charging by the electric double layer. Then the activation energy for a PCET step becomes:

12 $${G}_{{{{{{{{\rm{a}}}}}}}},i}^{\circ }={G}_{{{{{{{{\rm{a}}}}}}}},i}^{\circ }\left(U={U}_{i}^{{{{{{{{\rm{rev}}}}}}}},\circ }\right)+{\beta }_{i}{\Delta }_{i{{{{{{{\rm{R}}}}}}}}}{\Omega }^{\circ }(\sigma (U)),$$

where ${G}_{{{{{{{{\rm{a}}}}}}}},i}^{\circ }(U={U}_{i}^{{{{{{{{\rm{rev}}}}}}}},\circ })$ is the activation energy at the reversible potential ${U}_{i}^{{{{{{{{\rm{rev}}}}}}}},\circ }$ of the elementary step *i* to *i* + 1 and the ∘ indicates standard conditions (activities of 1, *pH* = 0). Δ_*i*R_Ω^∘^ is the difference in formation energies of two reaction intermediates *i* and *i* + 1, i.e. the reaction energy of that particular elementary step (the subscript R is used to differentiate it from the formation/adsorption energy which is defined relative to the empty slab). The meaning of *β*_*i*_ becomes clear when the reversible potential of each step is related at which:

13 $${\Delta }_{i{{{{{{{\rm{R}}}}}}}}}{\Omega }^{\circ }(\sigma ({U}_{i}^{{{{{{{{\rm{rev}}}}}}}},\circ }))=0$$

and therefore following Eq. (8):

14 $${U}_{i}^{{{{{{{{\rm{rev}}}}}}}},\circ }=-({\Delta }_{i{{{{{{{\rm{R}}}}}}}}}E(\sigma ({U}^{{{{{{{{\rm{rev}}}}}}}}}))+{\Delta }_{i{{{{{{{\rm{R}}}}}}}}}{E}^{{{{{{{{\rm{T,S,ZPE,\circ }}}}}}}}})/e$$

And therefore:

15 $$U-{U}_{i}^{{{{{{{{\rm{rev}}}}}}}},\circ }	=({\Delta }_{i{{{{{{{\rm{R}}}}}}}}}E(\sigma ({U}^{{{{{{{{\rm{rev}}}}}}}}}))+{\Delta }_{i{{{{{{{\rm{R}}}}}}}}}{E}^{{{{{{{{\rm{T,S,ZPE,\circ }}}}}}}}}+eU)/e \\ 	=\left({\Delta }_{i{{{{{{{\rm{R}}}}}}}}}{\Omega }^{\circ }(\sigma (U))-{\Delta }_{i{{{{{{{\rm{R}}}}}}}}}a\cdot (\sigma (U)-\sigma ({U}_{i}^{{{{{{{{\rm{rev}}}}}}}},\circ }))\right)/e,$$

where in the last term, we introduced again the grand potential which energetic contribution needs to be shifted to the right potential. With this expression, we can rewrite Eq. (12):

16 $${G}_{{{{{{{{\rm{a}}}}}}}},i}^{\circ }	={G}_{{{{{{{{\rm{a}}}}}}}},i}^{\circ }\left(U={U}_{i}^{{{{{{{{\rm{rev}}}}}}}},\circ }\right)+{\beta }_{i}e\left(U-{U}_{i}^{{{{{{{{\rm{rev}}}}}}}},\circ }\right)+{\beta }_{i}{\Delta }_{i}a\cdot \left(\sigma (U)-\sigma ({U}_{i}^{{{{{{{{\rm{rev}}}}}}}},\circ })\right)\\ 	={G}_{{{{{{{{\rm{a}}}}}}}},i}^{\circ }\left(U={U}_{i}^{{{{{{{{\rm{rev}}}}}}}},\circ }\right)+{\beta }_{i}e\left[1+1/e{\Delta }_{i}a{C}_{{{{{{{{\rm{gap}}}}}}}}}\right]\left(U-{U}_{i}^{{{{{{{{\rm{rev}}}}}}}},\circ }\right).$$

The first *β*_*i*_-term is the known term from reversible electrochemical kinetics formulated for each PCET step^36^. The last term depicts the charge stabilization of the transition state relative to the initial state. In experiment, usually an effective *β*^eff^ is observed, which can be defined by rewriting as^30^:

17 $${G}_{{{{{{{{\rm{a}}}}}}}},i}^{\circ }={G}_{{{{{{{{\rm{a}}}}}}}},i}^{\circ }\left(U={U}_{i}^{{{{{{{{\rm{rev}}}}}}}},\circ }\right)+{\beta }_{i}^{{{{{{{{\rm{eff}}}}}}}}}e\left(U-{U}_{i}^{{{{{{{{\rm{rev}}}}}}}},\circ }\right),$$

with the effective *β*^eff^ containing both contributions from the transferred electron and the double layer stabilization:

18 $${\beta }_{i}^{{{{{{{{\rm{eff}}}}}}}}}={\beta }_{i}\left[1+1/e{\Delta }_{i}a{C}_{{{{{{{{\rm{gap}}}}}}}}}\right]\quad .$$

We further used a pre-exponential factor of 10^13^ s^−1^ that was chosen based on the entropy-free pre-factor *k*_B_*T*/*h* (Boltzmann constant *k*_B_) that appears in transition state theory^124^ which was used by various kinetic CO_2_R studies^36,69^.

The above summarizes the calculation of barriers within CatMAP under standard conditions. Under non-standard conditions, e.g. *pH* > 0, the chemical potential of the initial state decreases, reducing the driving force Δ_*i*R_Ω by 0.0592 eV ⋅ *pH*. Assuming that the chemical potential of the transition state cannot be tuned by the pH^125^, this means that the activation energy changes by the same amount:

19 $${G}_{{{{{{{{\rm{a}}}}}}}},i}-{G}_{{{{{{{{\rm{a}}}}}}}},i}^{\circ }={\Delta }_{i{{{{{{{\rm{R}}}}}}}}}\Omega -{\Delta }_{i{{{{{{{\rm{R}}}}}}}}}{\Omega }^{\circ }=0.0592\,\,{{{{{{{\rm{eV}}}}}}}}\cdot pH.$$

#### Rationalization of kinetic barriers and model

In principle, kinetic parameters can be estimated e.g. via grand-canonical DFT^126,127^ or extrapolation methods^128,129^ using e.g. the nudged elastic band (NEB) method for localizing transition states. In practice, however, these simulations rely on a frozen electrolyte structure model from which activation energies on the 0K potential energy landscape are calculated. This generally neglects the dynamic nature of the electric double layer which can significantly contribute to the kinetic barrier. In addition, the use of hybrid implicit-explicit solvation methods in the grand-canonical schemes might lead to problems in capacitances and strong dependence on the interfacial water structure^43^. Due to these reasons, we decided instead to follow a semi-empirical approach to derive the kinetic parameters which we describe as follows.

In our previous work on CO_2_ reduction at the Au(211) facet, we have found from constant potential NEB calculations that the transition state of the CO_2_ adsorption and CO_2_ to *COOH steps lies very close to the final state for all potentials^30^. Following Eq. (12), this means that *β*_*i*_ ≈ 1 for these two steps. In addition, we found in the same work that the difference between activation energy and reaction energy, i.e. ${G}_{{{{{{{{\rm{a}}}}}}}},i}^{\circ }(U)-{\Delta }_{i{{{{{{{\rm{R}}}}}}}}}{\Omega }^{\circ }(\sigma (U))\,\approx \,0$ at all voltages, so that ${G}_{{{{{{{{\rm{a}}}}}}}},i}^{{{{{{{{\rm{rev}}}}}}}},\circ }({{{{{{{\rm{U}}}}}}}})\,\approx \,0$. For simplicity, we assumed that these conclusions hold also for all other considered metal surfaces, an approximation that has to be verified by future simulations. In line with our settings, a recent work has suggested that the transition state of the *CO_2_ to *COOH step lies close to *COOH with no additional kinetic barrier, but proposed an additional barrier for CO_2_ adsorption which we did not consider here^130^.

For the *COOH to *CO step, it is expected that the transition state is in the center of the initial and final states, due to the Tafel slope from previous experiments being 42 mV/dec. A value of 40 mV/dec is expected if the 2nd PCET is rate-limiting and ${\beta }_{i}^{{{{{{{{\rm{eff}}}}}}}}}\,\approx \,0.5$^40^. This corresponds to a value of *β*_*i*_ = 0.40 for Au(211) derived from Eq. (18) and the charging parameters that we calculated. For other metals, there can be significant changes in the Tafel slope, since high coverages of CO are expected on the strongly CO-adsorbing metals, which will change the kinetic equation that determines the Tafel slope. The charge-transfer coefficient is in principle more likely to be similar, but still, significant changes could occur. For simplicity in this work, we, however, ignored such variations in line with other previous works^36^. With the availability of more accurate means for electrochemical barriers, these assumptions should be tested again in future works.

The remaining free parameter is the activation energy at the reversible potential of the *COOH to *CO step. According to our previous collaborative experimental-theoretical work, this step becomes rate-limiting on Au at pH 6.8 at potentials larger than −0.7 V vs. SHE. At all potentials more negative than that CO_2_ adsorption is rate-limiting. We thus decided to tune this parameter to match the voltage at which the rate-limiting step changes with our model and by that obtained a value of ${G}_{{{{{{{{\rm{a}}}}}}}},3}^{\circ }(U={U}_{i}^{{{{{{{{\rm{rev}}}}}}}},\circ })=0.15$ eV. Choosing this value gives exactly the correct rate-limiting step transition voltage. See also Supplementary Materials Fig. S6 for the effect of this barrier on the rate-limiting step regions. Again, we assume that this value is constant across all metals again following the previous works^36^. This value might be at the low end of the error bar, since the *CO_2_ to *COOH step starts playing a role with this setting for the Au surfaces (cf. Supplementary Materials Fig. S10) which has not been observed experimentally^30^. With a slightly higher value, the importance of this step reduces (cf. Supplementary Materials Fig. S6). Also recent theoretical studies suggest a bit higher barrier^130^.

Lastly, the activation barrier for CO desorption was set to zero, following previous studies which generally found small kinetic barriers on Ru and Pt^131,132^.

#### Pressures

All micro-kinetic models were evaluated for a CO_2_ pressure of 1 bar and CO pressure of 0. Further, we set the H_2_O pressure to the vapor pressure in equilibrium with the liquid phase *P*_vap_ = 3.5 kPa, roughly the experimental value at room temperature^133^ to convert the gas phase chemical potential into a liquid phase chemical potential ^30^.

## Supplementary information

Supplementary Information

Description of additional supplementary files

Supplementary Data

## Data Availability

All other data is available on request from the author. Source data are provided with this paper.

## Source data

Source Data
