# Supplementary material for: The importance of a charge transfer descriptor for screening potential CO2 reduction electrocatalysts
Source: Nat Commun. 2023 May 5;14:2598. doi: 10.1038/s41467-023-37929-4 (PMC10162986; doi:10.1038/s41467-023-37929-4)
Supplement: Supplementary file 1 — Supplementary Information [file 41467_2023_37929_MOESM1_ESM.pdf]

The importance of a charge transfer descriptor for screening potential CO_2 reduction electrocatalysts

Stefan Ringe*
Department of Chemistry, Korea University, Seoul 02841, Republic of Korea

* sringe@korea.ac.kr

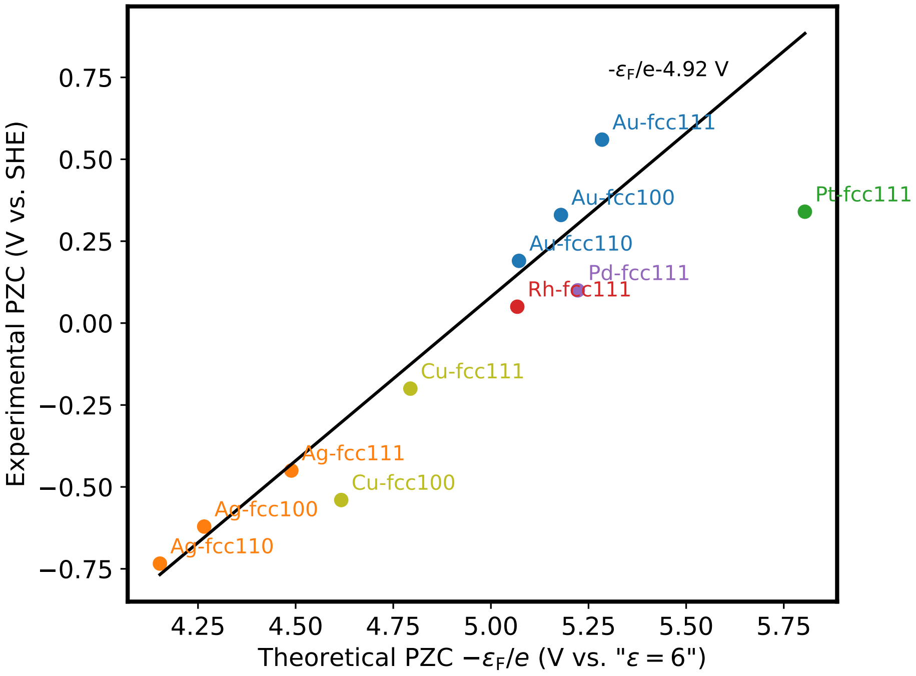

FIG. S1: Linear correlation of experimentally measured PZC vs. SHE in NaF electrolyte from ref. [13] with theoretical PZC vs. $\varepsilon^{\mathrm{b}} = 6$ implicit solvent. The two properties are by a constant shift of 4.92 V. Source data are provided as a Source Data file.

adsorbate	metal	facet	supercell	$\Delta_i b$ (10^4 eV cm$^4/\mu$C^2)	$\Delta_i a$ (10^2 eV cm$^2/\mu$C)	$\Delta_i E_0$ (eV)
CO	Ag	(100)	4x4	-0.18	-0.47	-0.19
CO	Ag	(111)	4x4	-1.47	-0.68	-0.04
CO	Al	(111)	4x4	-1.62	0.59	-0.33
CO	Au	(100)	4x4	0.15	-1.23	-0.32
CO	Au	(111)	4x4	-1.75	-1.46	-0.08
CO	Au	(211)	3x4	-2.37	-1.11	-0.38
CO	Cu	(100)	4x4	0.56	-0.18	-0.66
CO	Cu	(211)	3x4	-1.13	0.06	-0.75
CO	Pd	(100)	4x4	0.12	0.29	-1.22
CO	Pd	(111)	4x4	-0.31	0.03	-1.17
CO	Pd_3Au	(111)	4x4	-5.2	-0.43	-1.01
CO	Pt	(100)	4x4	-0.47	-0.19	-1.66
CO	Pt	(111)	4x4	-0.41	-0.61	-1.5
CO	Rh	(100)	4x4	-0.66	0.86	-1.65
CO	Rh	(111)	4x4	-0.39	0.58	-1.65
CO	Rh	(211)	3x4	-1.38	1.41	-1.75
CO_2	Ag	(111)	4x4	-2.97	3.69	0.85
CO_2	Ag	(211)	3x4	-4.06	3.32	0.68
CO_2	Al	(111)	4x4	-11.64	3.63	0.38
CO_2	Au	(211)	3x4	-5.44	3.18	0.85
CO_2	Cu	(111)	4x4	-1.06	3.59	0.92
CO_2	Cu	(211)	3x4	-1.81	3.46	0.48
CO_2	Pd	(111)	4x4	-1.96	2.45	0.26
CO_2	Pd	(211)	3x4	-2.52	2.79	0.03
CO_2	Pd_3Au	(111)	4x4	-0.52	2.65	0.42
CO_2	Pt	(100)	4x4	-2.92	2.26	0.37
CO_2	Pt	(211)	3x4	-3.51	2.59	0.01
CO_2	Rh	(100)	4x4	5.34	3.07	-0.07
CO_2	Rh	(111)	4x4	0.19	3.24	0.1
CO_2	Rh	(211)	3x4	-3.21	3	-0.25
COOH	Ag	(100)	4x4	-0.91	1.39	0.68
COOH	Ag	(111)	4x4	-3.13	0.95	0.87
COOH	Al	(111)	4x4	-0.21	1.86	0.16
COOH	Au	(100)	4x4	0.07	0.07	0.54
COOH	Au	(111)	4x4	-3.7	-0.81	0.68
COOH	Au	(211)	3x4	-2.8	0.18	0.52
COOH	Cu	(100)	4x4	0.42	0.51	0.41
COOH	Cu	(211)	3x4	-0.19	0.95	0.28
COOH	Pd	(100)	4x4	-1.24	-0.2	0.01
COOH	Pd	(111)	4x4	-4.02	-0.78	0.01
COOH	Pd_3Au	(111)	4x4	-2.37	-0.66	0.14
COOH	Pt	(100)	4x4	-0.18	-0.3	-0.31
COOH	Pt	(111)	4x4	-1.99	-0.95	-0.29
COOH	Rh	(100)	4x4	-1.25	0.16	-0.19
COOH	Rh	(111)	4x4	-2.56	0.32	-0.11
COOH	Rh	(211)	3x4	-1.14	0.72	-0.33

TABLE S1: Surface-charge density dependency parameters for all calculated facets and adsorbates. The parameters were obtained from surface-charge-dependent DFT calculations and quadratic interpolation of the surface charge dependence. The quadratic order term is ignored in this work. Only those cases are shown in the table for which the surface charge dependence was explicitly calculated via DFT and interpolation.

property (unit)	α (unit/eV)	β (unit/eV)	γ (unit)	R^2
$\Delta_{\mathrm{CO_2}}a$ (eV cm^2/μC)	0.0072	0.0015	0.0674	0.757
$\Delta_{\mathrm{COOH}}a$ (eV cm^2/μC)	0.016	-0.0016	0.0801	0.86
$\Delta_{\mathrm{CO}}a$ (eV cm^2/μC)	0.0129	-0.0134	0.0504	0.96
$\Delta_{\mathrm{CO_2}}E_0$ (eV)	-0.4010	0.65	-0.9575	0.941
$\Delta_{\mathrm{COOH}}E_0$ (eV)	-0.0432	0.6063	0.5214	0.905
$\Delta_{\mathrm{CO}}E_0$ (eV)	0.0	1.0	0.0	1.0

TABLE S2: Multi-linear scaling relations found for all properties, according to the relation property $= \alpha \cdot \epsilon_{\mathrm{F}}(\mathrm{vs.}\ \varepsilon^{\mathrm{b}} = 6) + \beta \cdot \Delta_{\mathrm{CO}}E_0 + \gamma$

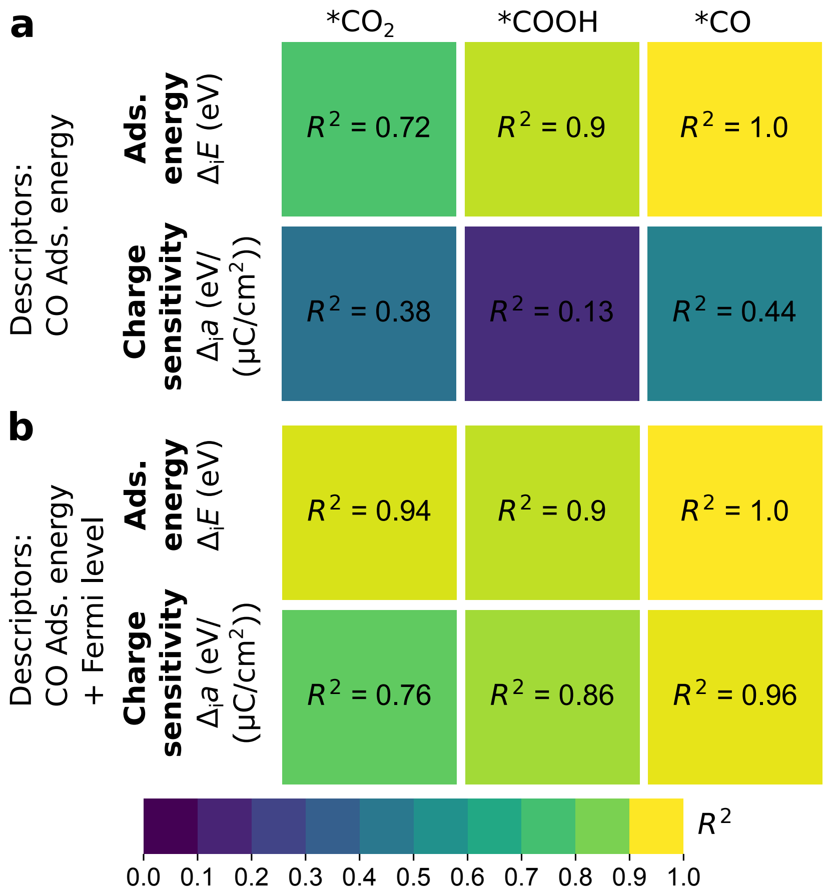

FIG. S2: Linear regression analysis of adsorption energies and charge sensitivity parameters. The figure shows the R^2 coefficient obtained by linearly correlating the adsorption energies of $^*CO_2$, *COOH and *CO with the adsorption energy of *CO (a) and both the adsorption energy of *CO and the Fermi level (b, multi-linear regression). Source data are provided in Fig. Table S1 and S2.

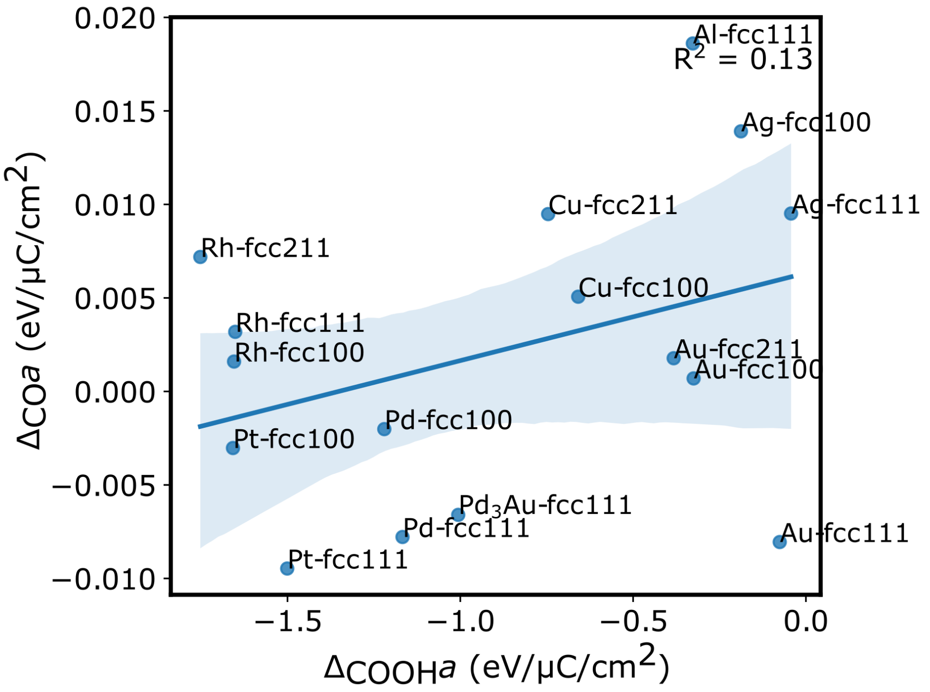

FIG. S3: Missing linear correlation of charging parameters of *COOH and *CO intermediates that is one of the reasons for the breaking of the scaling relations. Source data are provided in Table S1.

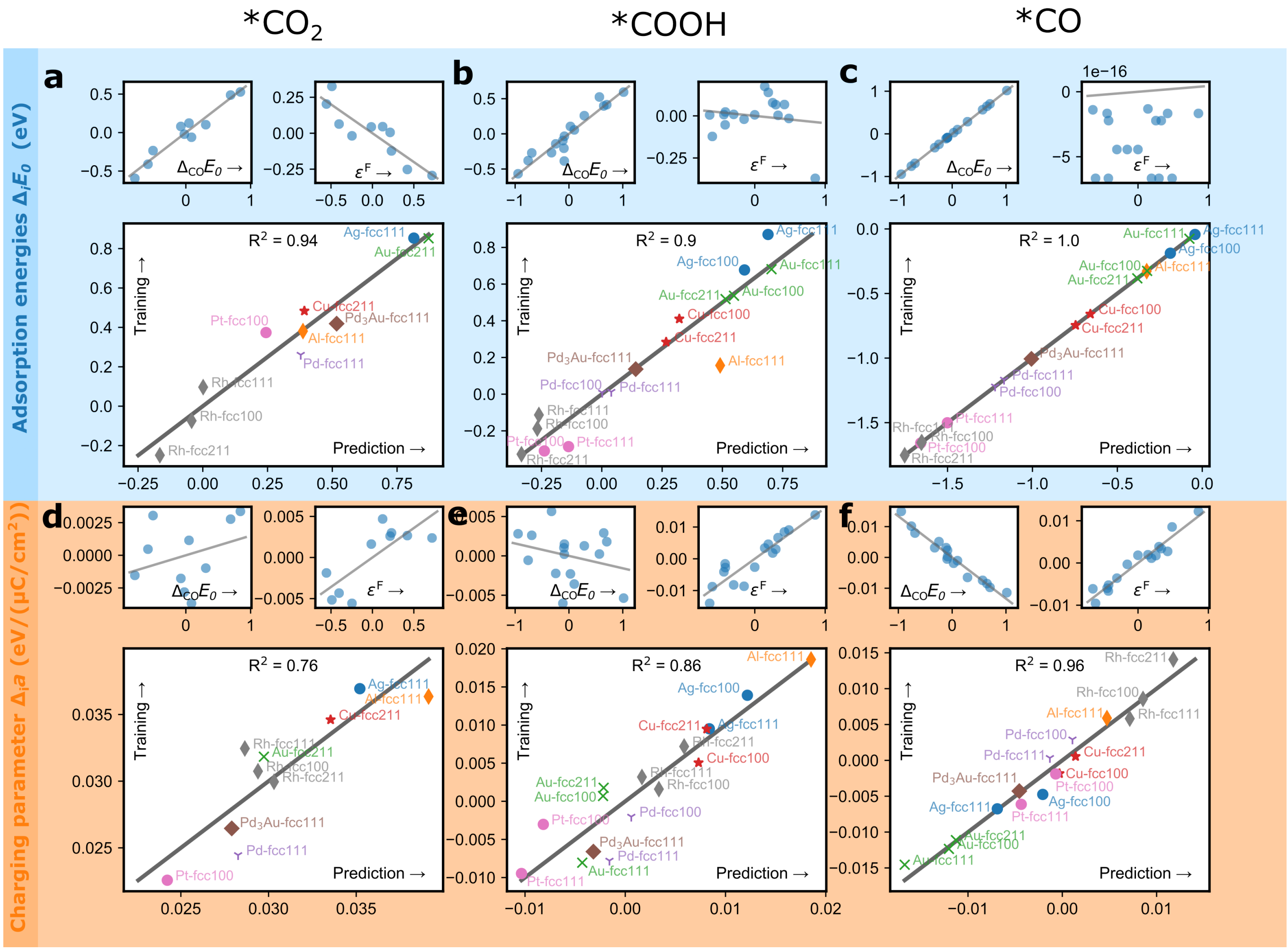

FIG. S4: Multi-linear regression of all intermediate adsorption energies and charging parameters with the CO adsorption energy and the Fermi level. The big panels show the correlation of prediction of the property and reference value, the small panels show the partial linear regression plots, highlighting the separate correlation of the two descriptors with the property of interest. Source data are provided in Table S1 and S2.

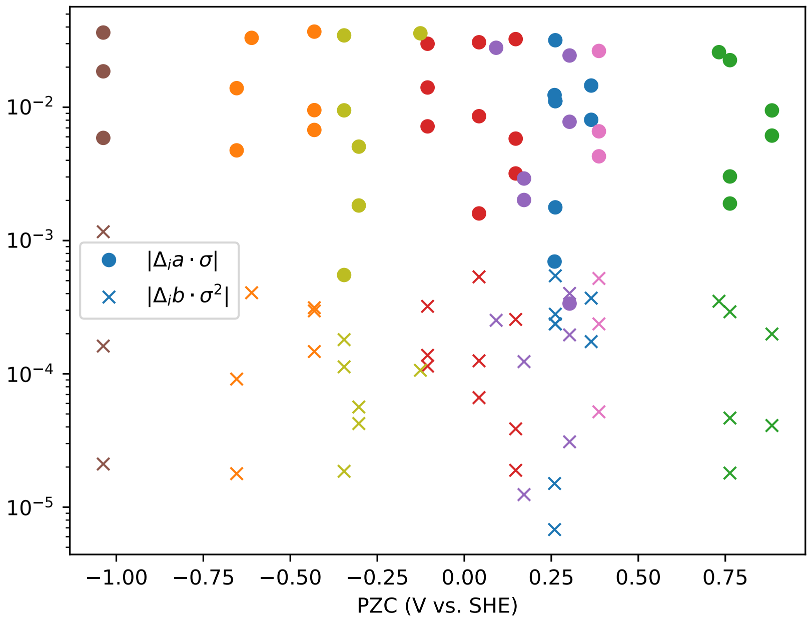

FIG. S5: Plot of the linear and quadratic contribution (absolute value) to the adsorption energy of *CO_2, *COOH, and *CO for all metal surfaces at -1 V vs. SHE. The color code of metals is the same as for the volcano plots. Source data are provided as a Source Data file.

S1. PRACTICAL NUMERICAL EXAMPLE OF FORMATION ENERGY CALCULATION FOR CLARIFICATION

To clarify the application of all energetic conversion, I give one example. It is searched for the free energies of all intermediates and transition states at -1.0 V vs. SHE as well as pH = 6.8 and $\Delta_{\mathrm{CO}}E = -0.509$ eV and $U^{\mathrm{PZC}} = 0.108$ V, the values are arbitrary points in the contour plot. First, the voltage is converged to a surface charge density, using:

$$\sigma = C_{\mathrm{gap}} \cdot (U - U^{\mathrm{PZC}}) = 20\ \mu\mathrm{F/cm}^2 \cdot (-1\ \mathrm{V} - 0.11\ \mathrm{V}) = -22.2\ \mu\mathrm{C/cm}^2 \tag{S1}$$

Now, the Fermi level vs. $\varepsilon^{\mathrm{b}} = 6$ is calculated from the relation in Fig. S1:

$$\epsilon^{\mathrm{F}} = -e(U^{\mathrm{PZC}} + 4.92\ \mathrm{V}) = -5.03\ \mathrm{eV} \tag{S2}$$

Now, the formation energies and charge sensitivity parameter can be calculated according to the relations in Table S2, as an example given here for *COOH:

$$\begin{aligned}\Delta_{\mathrm{COOH}}E_0 &= \alpha \cdot \epsilon^{\mathrm{F}} + \beta \cdot \Delta_{\mathrm{CO}}E + \gamma = 0.0432 \cdot 5.03\ \mathrm{eV} + 0.6063 \cdot (-0.509\ \mathrm{eV}) + 0.5214\ \mathrm{eV} = 0.43\ \mathrm{eV}\\ \Delta_{\mathrm{COOH}}a &= \alpha \cdot \epsilon^{\mathrm{F}} + \beta \cdot \Delta_{\mathrm{CO}}E + \gamma\\ &= -0.016 \cdot 5.03\ \mathrm{eV}/(\mu\mathrm{C/cm}^2) - 0.0016 \cdot (-0.509\ \mathrm{eV}/(\mu\mathrm{C/cm}^2)) + 0.0801\ \mathrm{eV}/(\mu\mathrm{C/cm}^2)\\ &= 4.34 \cdot 10^{-4}\ \mathrm{eV}/(\mu\mathrm{C/cm}^2)\end{aligned} \tag{S3}$$

With this, the formation energy at the applied potential can be calculated as (Eq. 11):

$$\Delta_{\mathrm{COOH}}E^U(U) = \Delta_{\mathrm{COOH}}E_0 + \Delta_{\mathrm{COOH}}a \cdot \sigma(U) = 0.43\ \mathrm{eV} + 4.34 \cdot 10^{-4}\ \mathrm{eV}/(\mu\mathrm{C/cm}^2) \cdot (-22.2\ \mu\mathrm{C/cm}^2) = 0.42\ \mathrm{eV} \tag{S4}$$

After this, finite temperature and ZPE corrections are added to each intermediate, as well as the gas phase corrections (0.33 eV for CO_2, 0.09 eV for H_2). In addition, pH and potential corrections according to the CHE model. Following

Eq. 8, we get exemplarily for the formation of *COOH from $CO_{2,g}$:

$$\Delta_{\mathrm{COOH}}\Omega(U) = \Delta_{\mathrm{COOH}}E(\sigma(U)) + \Delta_{\mathrm{COOH}}E^{\mathrm{T,S,ZPE,\circ}} + eU + 0.0592\ \mathrm{eV}\cdot pH \quad \text{(S5)}$$

$$= 0.42\ \mathrm{eV} + 0.87\ \mathrm{eV} - 1.0\ \mathrm{eV} + 0.0592\ \mathrm{eV}\cdot 6.8 - 0.33 - 0.09/2 = 0.36\ \mathrm{eV} \quad . \quad \text{(S6)}$$

The kinetic barriers are calculated by scaling the reaction energies from one to the next elementary step with the corresponding β coefficient and adding the activation energy at the reversible potential, following Eq. 12. All energies are plugged into a kinetic model and the TOF is determined from solving it in the steady-state approximation.

S2. MICRO-KINETIC MODELING

Micro-kinetic modeling was performed for a grid of 50×50 points that span the PZC-CO adsorption energy space. At each point, first, the linear correlation map was used to predict all formation energies and charging coefficients. Internally within CatMAP the energies were modified by voltage and charging contributions as detailed in the equations in the methods section of the main manuscript.

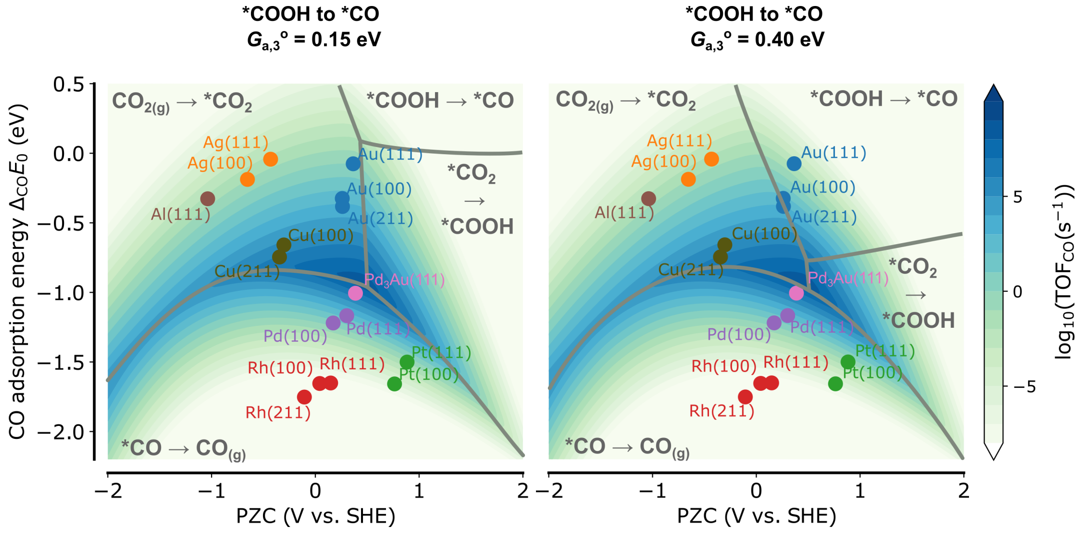

FIG. S6: Effect of *COOH to *CO barrier at the reversible potential $G^{\circ}_{a,3}$ at pH = 6.8 and -1.0 V vs. SHE. Source data are provided as a Source Data file.

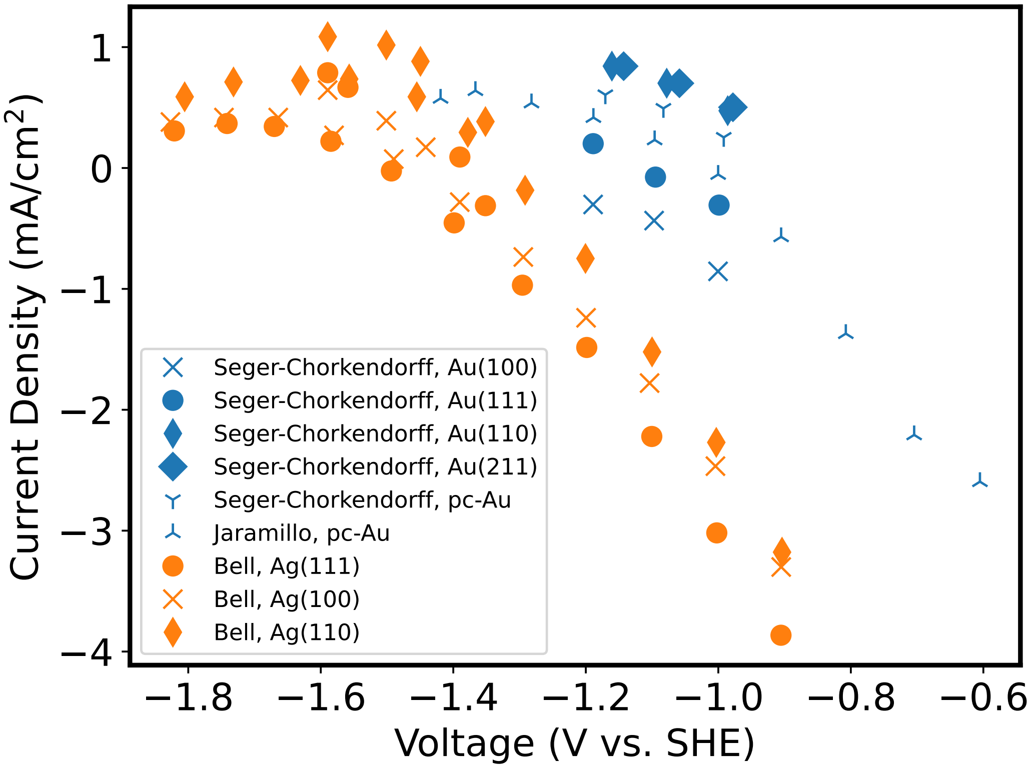

FIG. S7: Comparison of experimental data for the CO partial current density from electrochemical CO_2 reduction in an H-cell. References: Hori[5], Jaramillo,[12] Bell,[1] and Seger-Chorkendorff.[9]

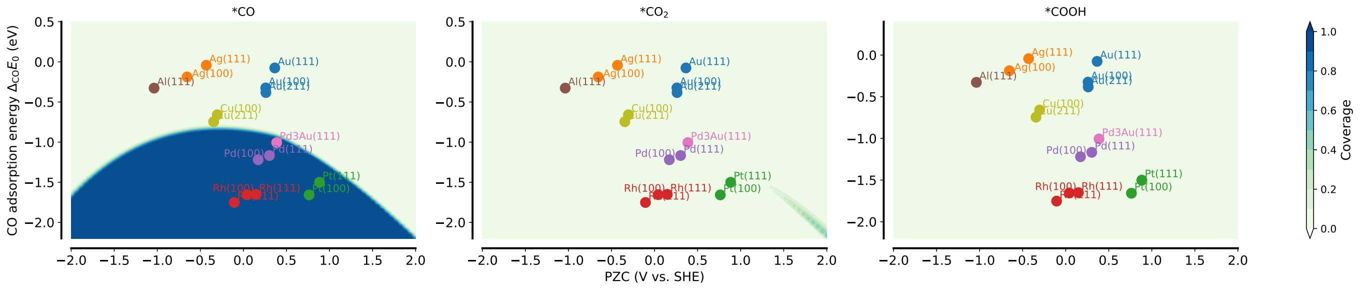

FIG. S8: Coverages of the three intermediates as a function of the newly determined descriptor space for micro-kinetic simulations at pH = 6.8 and -1.0 V vs. SHE. Source data are provided as a Source Data file.

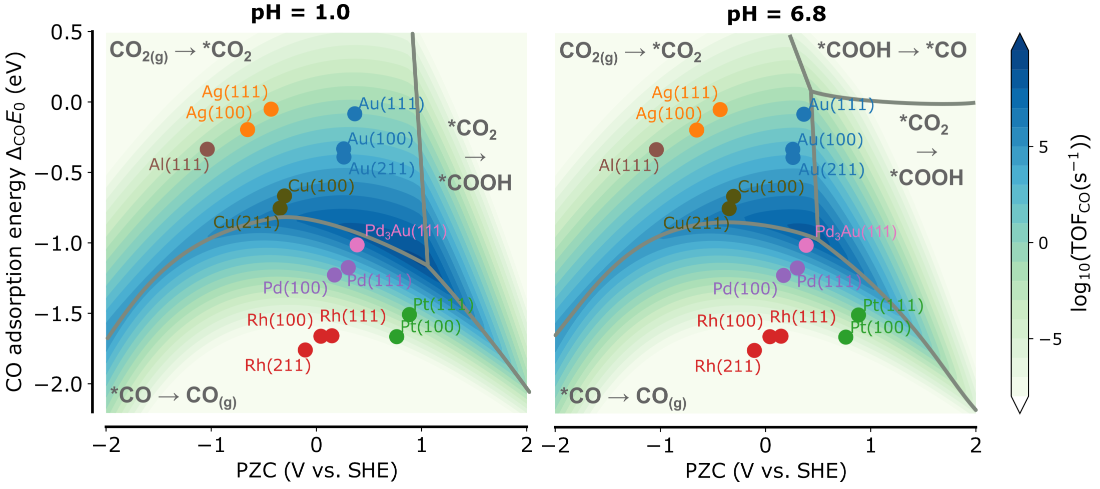

FIG. S9: pH effect on the CO_2 to CO activity volcano and rate-limiting step landscape. Left: pH = 1, right pH = 6.8, all at -1.0 V vs. SHE. Source data are provided as a Source Data file.

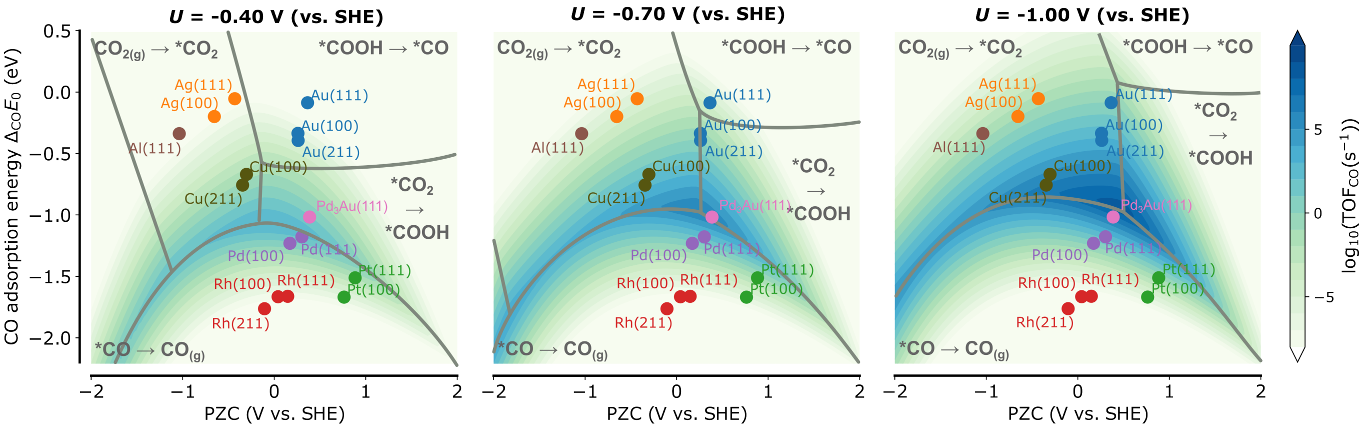

FIG. S10: Voltage effect on the CO_2 to CO activity volcano and rate-limiting step landscape. Left: $U = -0.4V$ (vs. SHE), center: $U = -0.7V$ (vs. SHE), right $U = -1.0V$ (vs. SHE), all at pH = 6.8. Source data are provided as a Source Data file.

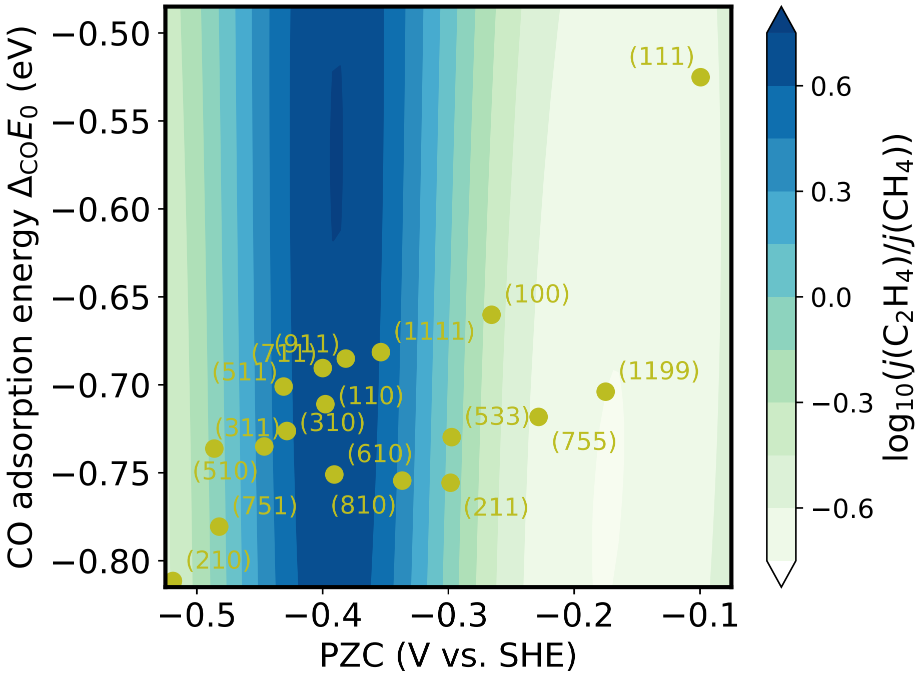

FIG. S11: **C_2H_4 vs. CH_4 product selectivity (log scale) from experimental Hori[3] data interpolated using a Gaussian process regression.** The data is shown as a function of the descriptor space determined in this work. For the Gaussian process regression, I used the scikit-learn package[11] and a radial basis function kernel with 0.01 V and 0.008 eV length scales matching the span of the data, as well as a regularization $\alpha = 0.1$ to account for possible experimental errors. Source data are provided as a Source Data file.

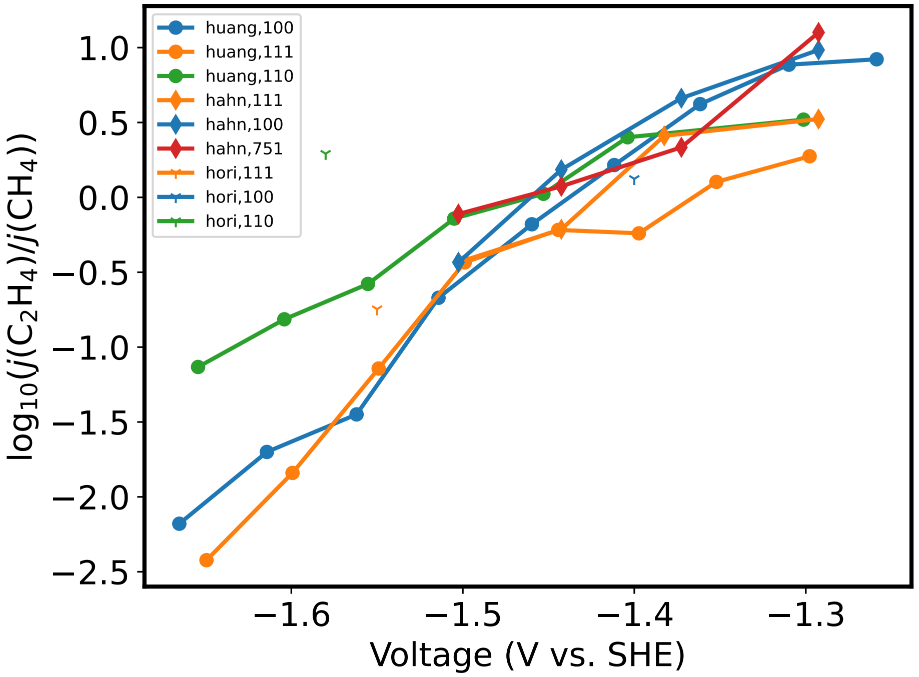

FIG. S12: **Potential-dependent C_2H_4 vs. CH_4 product selectivity (log scale) extracted from experimental H-cell CO_2RR data from huang,[6] hori,[3] and hahn.[2]** Source data are provided as a Source Data file.

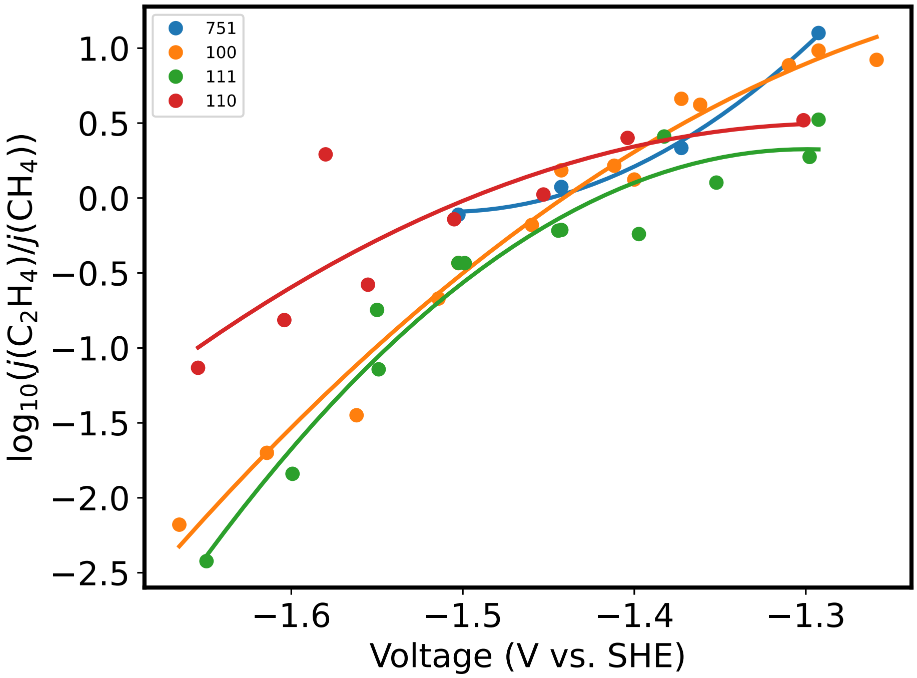

FIG. S13: **Potential-dependent C_2H_4 vs. CH_4 product selectivity (log scale) extracted from experimental H-cell CO_2RR data from huang,[6] hori,[3] and hahn[2] interpolated by a quadratic spline for each facet.** Source data are provided as a Source Data file.

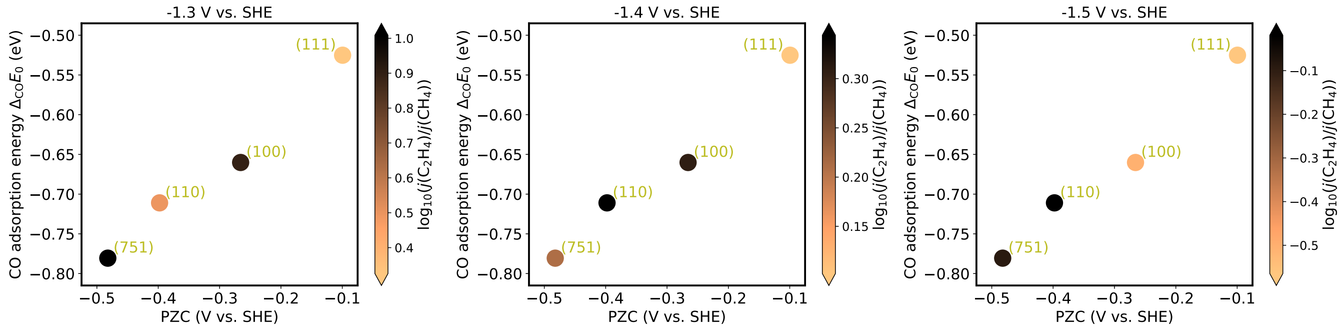

FIG. S14: **C_2H_4 vs. CH_4 product selectivity (log scale) extracted Fig. S13 at three potentials and plotted vs. the descriptors determined in this work.** Source data are provided as a Source Data file.

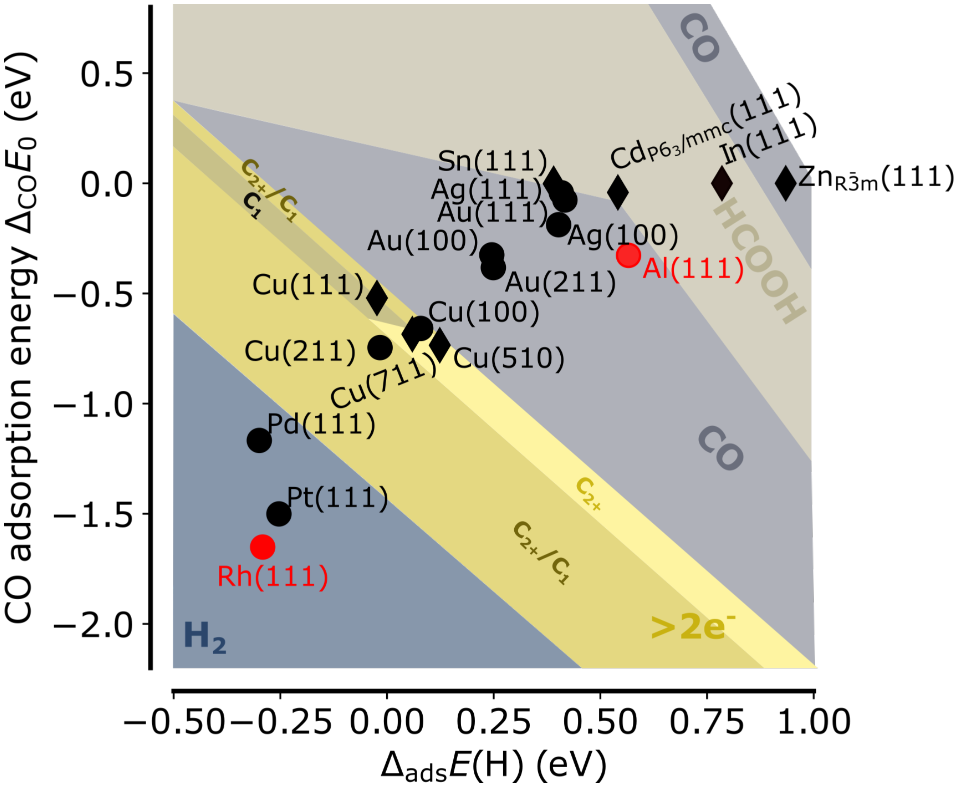

FIG. S15: **Product selectivity as a function of the conventional H and CO adsorption energy descriptors.** Main product selectivity in electrochemical CO_2RR as a function of descriptor space. The main selectivity was extracted from H-cell experiments at -1.4 V vs. SHE (pH = 6.8) using poly- and single-crystalline planar electrodes from the experimental work of Hori *et al.*[4] and others (Cu,[3] In,[10] Au,[7, 9, 12] Ag,[1] Sn,[8, 14] Zn,[7]). Black symbols refer to points where experimental selectivity data was available under these conditions, and red ones to those, where selectivity data was missing. Circles refer to the data points which were actually used for training our descriptor model and diamonds for those where only the descriptor values were evaluated. Source data are provided as a Source Data file.

S3. EXPERIMENTAL SELECTIVITY PLOTS

The main selectivity was extracted from H-cell experiments at -1.4 V vs. SHE (pH = 6.8) using poly-crystalline and single-crystalline planar electrodes from the experimental work of Hori *et al.*[4] and others (Cu,[2, 3, 6] In,[10] Au,[7, 9, 12] Ag,[1] Sn,[8, 14] Zn,[7]).

REFERENCES

[1] Clark, E. L., Ringe, S., Tang, M., Walton, A., Hahn, C., Jaramillo, T. F., Chan, K., and Bell, A. T. Influence of atomic surface structure on the activity of ag for the electrochemical reduction of CO2 to CO. *ACS Catal.* (Mar. 2019), 4006–4014.

[2] Hahn, C., Hatsukade, T., Kim, Y.-G., Vailionis, A., Baricuatro, J. H., Higgins, D. C., Nitopi, S. A., Soriaga, M. P., and Jaramillo, T. F. Engineering cu surfaces for the electrocatalytic conversion of CO2: Controlling selectivity toward oxygenates and hydrocarbons. *Proc. Natl. Acad. Sci. U. S. A. 114*, 23 (June 2017), 5918–5923.

[3] Hori, Y., Takahashi, I., Koga, O., and Hoshi, N. Electrochemical reduction of carbon dioxide at various series of copper single crystal electrodes. *J. Mol. Catal. A Chem. 199*, 1 (May 2003), 39–47.

[4] Hori, Y., Wakebe, H., Tsukamoto, T., and Koga, O. Electrocatalytic process of CO selectivity in electrochemical reduction of CO2 at metal electrodes in aqueous media, 1994.

[5] Hoshi, N., Kato, M., and Hori, Y. Electrochemical reduction of CO2 on single crystal electrodes of silver ag(111), ag(100) and ag(110). *J. Electroanal. Chem. 440*, 1 (Dec. 1997), 283–286.

[6] Huang, Y., Handoko, A. D., Hirunsit, P., and Yeo, B. S. Electrochemical reduction of CO2 using copper Single-Crystal surfaces: Effects of CO* coverage on the selective formation of ethylene. *ACS Catal. 7*, 3 (Mar. 2017), 1749–1756.

[7] Kuhl, K. P., Hatsukade, T., Cave, E. R., Abram, D. N., Kibsgaard, J., and Jaramillo, T. F. Electrocatalytic conversion of carbon dioxide to methane and methanol on transition metal surfaces. *J. Am. Chem. Soc. 136*, 40 (Oct. 2014), 14107–14113.

[8] Li, J., Jiao, J., Zhang, H., Zhu, P., Ma, H., Chen, C., Xiao, H., and Lu, Q. Two-Dimensional SnO2 nanosheets for efficient carbon dioxide electroreduction to formate. *ACS Sustainable Chem. Eng. 8*, 12 (Mar. 2020), 4975–4982.

[9] Mezzavilla, S., Horch, S., Stephens, I. E. L., Seger, B., and Chorkendorff, I. Structure sensitivity in the electrocatalytic reduction of CO2 with gold catalysts. *Angew. Chem. Int. Ed Engl.* (Jan. 2019).

[10] Noda, H., Ikeda, S., Oda, Y., Imai, K., Maeda, M., and Ito, K. Electrochemical reduction of carbon dioxide at various metal electrodes in aqueous potassium hydrogen carbonate solution. *Bull. Chem. Soc. Jpn. 63*, 9 (1990), 2459–2462.

[11] Pedregosa, F., Varoquaux, G., Gramfort, A., Michel, V., Thirion, B., Grisel, O., Blondel, M., Prettenhofer, P., Weiss, R., Dubourg, V., Vanderplas, J., Passos, A., Cournapeau, D., Brucher, M., Perrot, M., and Duchesnay, E. Scikit-learn: Machine learning in Python. *Journal of Machine Learning Research 12* (2011), 2825–2830.

[12] Ringe, S., Morales-Guio, C. G., Chen, L. D., Fields, M., Jaramillo, T. F., Hahn, C., and Chan, K. Double layer charging driven carbon dioxide adsorption limits the rate of electrochemical carbon dioxide reduction on gold. *Nat. Commun. 11*, 1 (January 2020), 33.

[13] Trasatti, S., and Lust, E. The potential of zero charge. In *Modern Aspects of Electrochemistry*, R. E. White and B. E. C. J. O'M. Bockris, Eds., vol. 33. KluwerAcademic/PlenumPublishers, New York, 1999.

[14] Yang, Z., Oropeza, F. E., and Zhang, K. H. L. P-block metal-based (sn, in, bi, pb) electrocatalysts for selective reduction of CO2 to formate. *APL Materials 8*, 6 (June 2020), 060901.
