# Supplementary material for: The importance of a charge transfer descriptor for screening potential CO2 reduction electrocatalysts
Source: Nat Commun. 2023 May 5;14:2598. doi: 10.1038/s41467-023-37929-4 (PMC10162986; doi:10.1038/s41467-023-37929-4)
Supplement: Supplementary file 2 — Description of additional supplementary files [file 41467_2023_37929_MOESM2_ESM.pdf]

<u>Description of additional supplementary files</u>

Supplementary Data file: This Supplementary Data zip file contains a text file in the json format which contains the input settings for all DFT calculations, the optimized geometry and the resulting energy. The file can be read by any text editor or by the Atomic Simulation Environment (ASE) database (https://wiki.fysik.dtu.dk/ase/ase/db/db.html) module.
